# Short-Term and Long-Term Biological Effects of Chronic Chemical Contamination on Natural Populations of a Marine Bivalve

**DOI:** 10.1371/journal.pone.0150184

**Published:** 2016-03-03

**Authors:** Marine Breitwieser, Amélia Viricel, Marianne Graber, Laurence Murillo, Vanessa Becquet, Carine Churlaud, Ingrid Fruitier-Arnaudin, Valérie Huet, Camille Lacroix, Eric Pante, Stéphane Le Floch, Hélène Thomas-Guyon

**Affiliations:** 1 Littoral Environnement et Sociétés (LIENSs), UMR 7266, CNRS-Université de La Rochelle, 2 rue Olympe de Gouges, F-17042, La Rochelle, Cedex 01, France; 2 Cedre, Centre de Documentation, de Recherche et d’Expérimentations sur les Pollutions Accidentelles des Eaux, 715 rue Alain Colas, CS 41836, Brest, Cedex 2, France; CINVESTAV-IPN, MEXICO

## Abstract

Understanding the effects of chronic chemical contamination on natural populations of marine organisms is complex due to the combined effects of different types of pollutants and environmental parameters that can modulate the physiological responses to stress. Here, we present the effects of a chronic contamination in a marine bivalve by combining multiple approaches that provide information on individual and population health. We sampled variegated scallops (*Mimachlamys varia*) at sites characterized by different contaminants and contamination levels to study the short and long-term (intergenerational) responses of this species to physiological stress. We used biomarkers (SOD, MDA, GST, laccase, citrate synthase and phosphatases) as indicators of oxidative stress, immune system alteration, mitochondrial respiration and general metabolism, and measured population genetic diversity at each site. In parallel, concentration of 14 trace metals and 45 organic contaminants (PAHs, PCBs, pesticides) in tissues were measured. Scallops were collected outside and during their reproductive season to investigate temporal variability in contaminant and biomarker levels. Our analyses revealed that the levels of two biomarkers (Laccase-type phenoloxidase and malondialdehyde) were significantly correlated with Cd concentration. Additionally, we observed significant seasonal differences for four of the five biomarkers, which is likely due to the scallop reproductive status at time of sampling. As a source of concern, a location that was identified as a reference site on the basis of inorganic contaminant levels presented the same level of some persistent organic pollutants (DDT and its metabolites) than more impacted sites. Finally, potential long-term effects of heavy metal contamination were observed for variegated scallops as genetic diversity was depressed in the most polluted sites.

## Introduction

Marine and coastal areas are affected by human activities at sea, on land and along the coast, that cause chronic and diffuse pollution (e.g., [1]). Fisheries, shipping, agriculture, industry and waste water treatment cause contamination of seawater, sediments and biota from the coastal areas by trace elements such as metals and radionuclides, and many organic pollutants such as pesticides and Polycyclic aromatic hydrocarbons (PAHs). In this context, the European Union's Marine Strategy Framework Directive (MSFD Dir. 2008/56/EC) was adopted in June 2008. It aims to achieve “Good Environmental Status” of marine environment by 2020 and to improve the conservation status of marine biodiversity. It is therefore crucial to find reliable methods to assess and monitor the chemical contamination of coastal areas and its effects on marine ecosystems. *In situ* experiments are paramount to achieving this goal, as they complement laboratory experiments by integrating the full complexity of natural systems and the simultaneous presence of all of contaminants in the environment. The chemical assessment of different contaminants (whether in the environment or in the tissues of marine organisms) is a fundamental first step in understanding the possible impact of pollution on marine species. However, this approach should be combined with biological approaches aimed at measuring the response of organisms to environmental insults. Indeed, biological or biochemical indicators provide information on the bio-availability of contaminants and the integration of the effects of multiple exposures over time. They offer a very effective and sensitive early warning system of longer-term negative effects. For example, biomarker responses reflect defense mechanisms that might be affected by environmental contaminants [2–6].

Marine bivalve mollusks are widely accepted as useful sentinels of chemical contamination in seawater because they are sedentary, filter great volumes of seawater and accumulate pollutants in high concentrations [7]. An increasing number of biomarkers for the detection of early biological response to cellular stress response signals generated by pollution are developed in bivalves [8]. The variegated scallop, *Mimachlamys varia*, appears as a potential sentinel species because it responds to specific biological criteria: sedentariness, ease of collection (it can be found on the intertidal zone), large distribution range (from the North Sea to the south east of the Atlantic Ocean and in Mediterranean sea) and potential of contaminants incorporation [9,10]. Moreover, its relevant bioaccumulation rate of contaminants makes it a very promising species for biomonitoring studies, with the digestive gland showing higher metal concentrations than other tissues such as the adductor muscle [11–14]. In that perspective, a recent *in situ* study on *M*. *varia* evaluated the responsiveness of several biomarkers that are commonly used for biomonitoring [6].

In the present paper, we evaluate the effects of chronic chemical contamination in a marine bivalve by combining multiple approaches that provide information on individual and population health. Samples of *M*. *varia* were collected on the Atlantic coast of France from three sites exposed to inorganic contamination and compared to a reference site, which was less contaminated [6,11,13]. The concentrations of 14 trace elements (Ag, As, Cd, Co, Cr, Cu, Fe, Mn, Ni, Pb, Se, Sn, V and Zn) were measured in the digestive glands of *M*. *varia*, since many of these metals are considered as tracers of anthropogenic inputs in coastal waters (e.g., [15]). Furthermore 45 organic contaminants were assayed in the same organ or the whole soft part of *M*. *varia*. Among them, organochlorine pesticides, such as dichlorodiphenyltrichloroethane (DDT) and its metabolites, aldrin, dieldrin and lindane are considered as POPs (Persistent Organic Pollutants), toxic, bioaccumulative and mutagenic [16]. PAHs, including pyrene, fluoranthene and naphthalene, considered as priority pollutants [1] and polychlorobiphenyl (PCB 153, 180) widely used in transformers or as lubricants, were also measured. Analysis of other seawater micropollutants such as radionuclides that can alter oxidative stress and bioaccumulate in marine bivalves (e.g. [17]) was beyond the scope of this study.

In parallel, six different biomarkers were used to monitor the modulation of oxidative stress, immune system alteration, mitochondrial respiration and general metabolism of *M*. *varia*. We relied on several biomarkers as they respond to complex mixtures of pollutants, which are expected for ecotoxicology studies conducted *in situ* [5–6]. Variations in both contaminant levels and biomarker responses were analyzed as a function of the season and the location to assess the potential of *M*. *varia* as a biomonitoring species.

**Fig 1:**
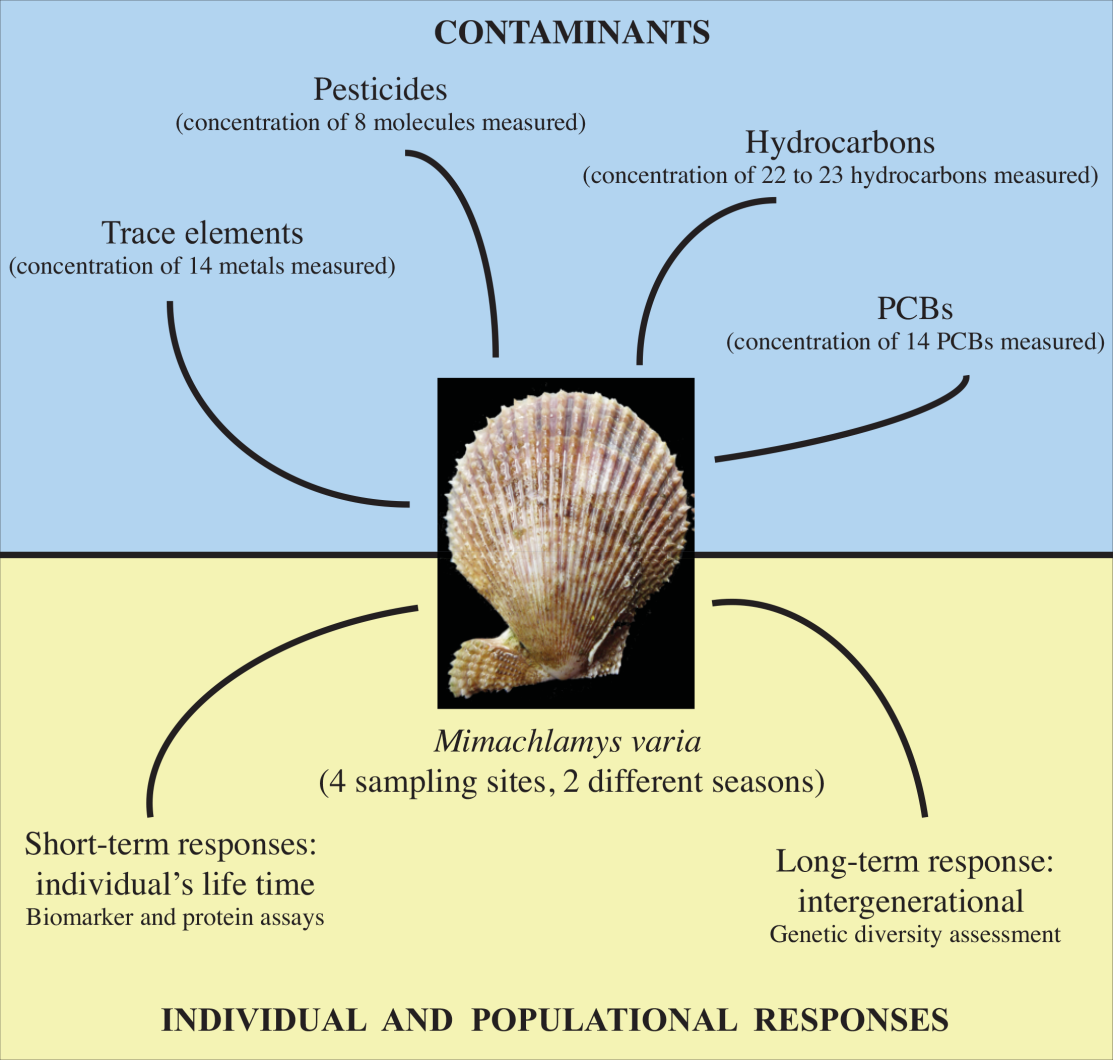
Integrative approach undertaken in this study to assess short- and long-term effects of a chronic chemical contamination on natural populations of the marine bivalve *Mimachlamys varia*. Photo credit: Thierry Guyot.

One important consideration to keep in mind in ecological studies is ensuring that the same taxon is being investigated throughout the experiment and when comparisons are made with previous studies. A species validation step is crucial when studying natural populations *in situ*, and particularly in the marine environment where difficulties in taxonomic assignment are exacerbated (see [18]). In this study, we relied on genetic tools to verify the species identity of collected scallops using a DNA barcoding approach [19]. We also used these genetic data to assess potential long-term (*i*.*e*., intergenerational) effects of a chronic chemical contamination on *M*. *varia* by estimating genetic diversity at each sample site. Our integrative, multi-time scale approach is illustrated in Fig 1.

To the best of our knowledge, there is no previous information about the bioaccumulation of organic pollutants in *M*. *varia* and no previous study assessing the effects of chemical pollutants on the genetic diversity of its populations. Indeed, previous ecotoxicological studies on *M*. *varia* in this region have focused on trace metal concentration bioaccumulation processes and correlations with biomarkers. Our goal was to obtain a more comprehensive evaluation of contaminants impacting these organisms, notably by including analyses of organic pollutants, which may be particularly relevant in our sampling areas given the influence of large effluents that may carry pesticides. Thus, this study provides a novel, integrative ecotoxicological assessment by investigating the links between two types of pollutants (trace metals and organic contaminants), biomarkers, and population genetic diversity.

## Materials and Methods

Collection of the marine bivalve *M*. *varia* was authorized by the French Ministère de l'Ecologie, du Développement Durable et de l'Energie (Direction interrégionale de la mer Sud-Atlantique) at the four sampling sites: Loix-en-Ré, Les Palles, Port-Neuf and Les Minimes (France). These sampling sites were not private or protected. Our field study did not involve an endangered or protected species.

### 1. *In situ* sampling and preparation of biochemical material

**Fig 2:**
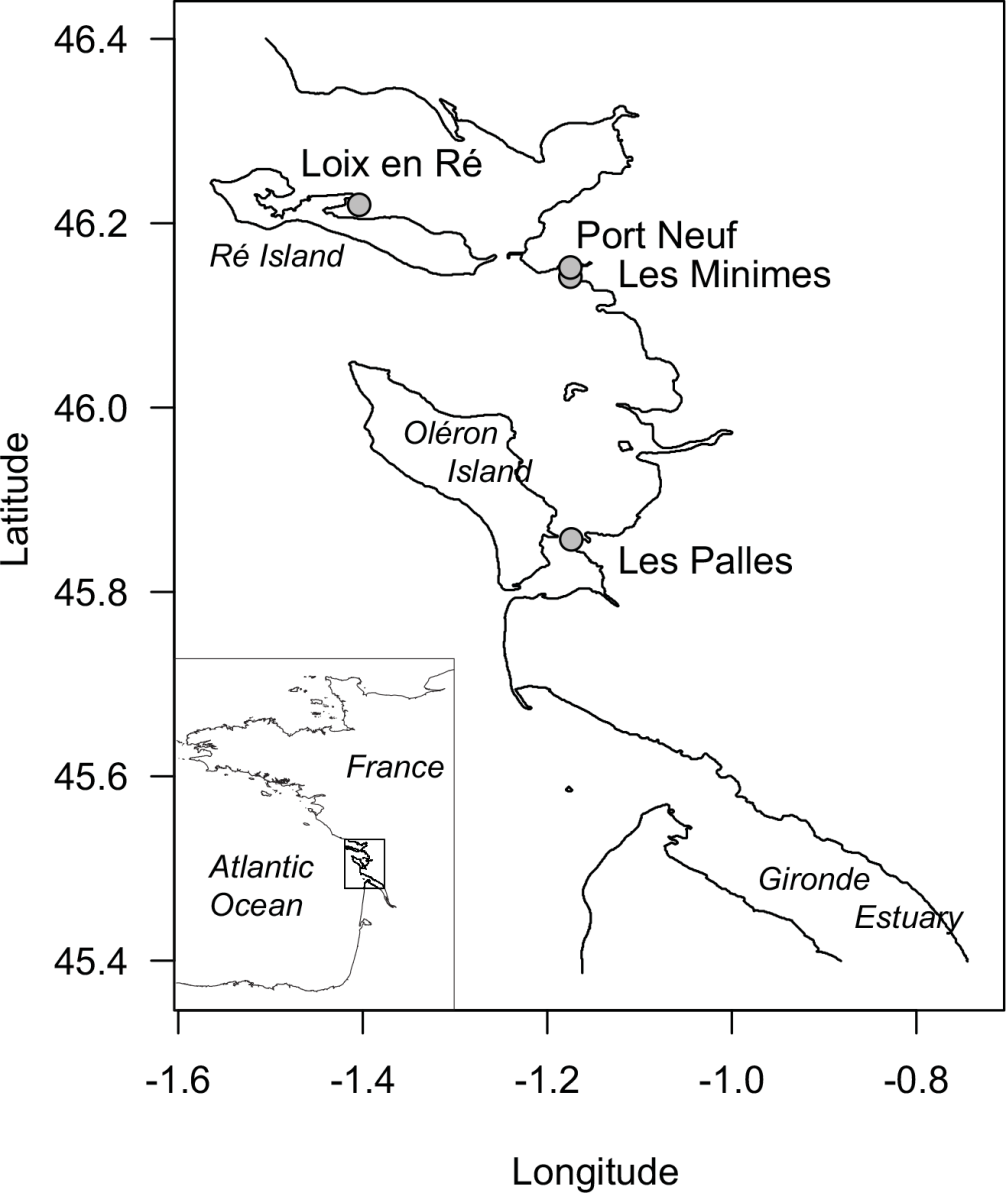
Location of the four sampling areas along the French Atlantic coast: Loix-en-Ré (Ré Island), Les Minimes (near sailing harbour), Port-Neuf (near water treatment of La Rochelle), Les Palles (near the mouth of the Charente estuary).

**Fig 3:**
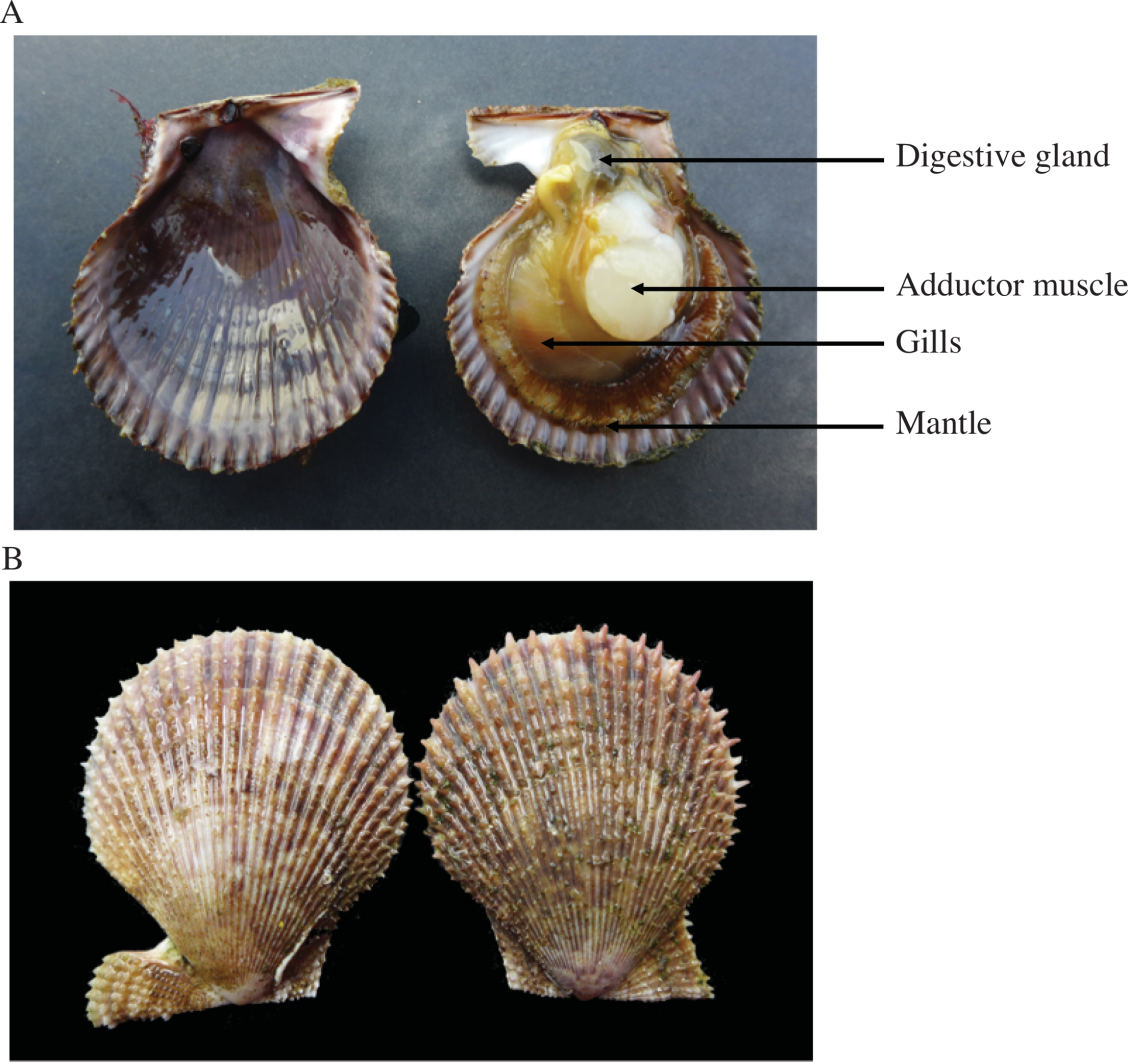
Photo showing the anatomy of the scallop *Mimachlamys varia* with (A) soft parts inside of the shell (digestive gland, adductor muscle, gills and mantle) and (B) outside of the shell. Photo credit: Thierry Guyot.

Sampling was conducted simultaneously on four sampling sites in March and September 2014 before and at the end of *M*. *varia*’s reproductive season, respectively [20]. Sampling occurred at low tide in the infralittoral zone at four sites along the French Atlantic coast (Charente Maritime; Fig 2): Loix-en-Ré (reference site), Port-Neuf (near the water treatment plant of La Rochelle), Les Minimes (near sailing harbour) and Les Palles (near the mouth of the Charente estuary). Ten to thirty scallops (Fig 3; adult height >35 mm) were collected at each sampling site. Table 1 summarizes how sampled individuals were either used for a single methodology or for multiple analyses. The preparation of biochemical material is described in S1 Appendix.

**Table 1:** Summary of our sampling design. Analyses of two types of chemical contaminants (trace metals and organic pollutants), biomarkers, phosphatases and genetic data were conducted on natural populations of variegated scallops (*Mimachlamys varia*) sampled at four sites. Analyses listed under the same “Analysis group” were conducted on the same variegated scallop individuals. All analyses but one were performed for each individual (sample size per site is shown in parentheses). The remaining analysis was conducted on a pool of 10 individuals per site. The type of tissue that was analyzed is indicated in the last column.

Analysis	Analysis group	Type of measurement: individual versus pooled samples	Analyzed tissues
SOD, LAC, GST, MDA	A	individual (n = 10 / site)	digestive gland
Citrate synthase	A	individual (n = 10 / site)	adductor muscle
Trace element analysis	A	individual (n = 10 / site)	digestive gland
Genetic analysis	A	individual (n = 30 / site)	adductor muscle
Phosphatase assay	B	1 pool of 10 individuals / site	adductor muscle + digestive gland
Pesticides, hydrocarbons, PCBs analyses	C	individual (n = 5<n<10 / site)	digestive gland

### 2. Species validation

We used a genetic approach to validate the species identity of collected scallops. For each of the four sites, twenty scallops collected in March and ten scallops collected in September were used for genetic analyses. Genomic DNA was extracted from approximately 15 mg of muscle tissue using the NucleoSpin^®^ Tissue kit (Macherey-Nagel EURL, Hoerdt, France) following the manufacturer’s protocol. Species identification was performed using a portion of the mitochondrial gene coding for cytochrome *c* oxidase I (*cox1*). This gene is commonly used in DNA barcoding studies as it is conserved across taxonomic groups and generally allows distinguishing closely related species [19]. The methodology for PCR reactions, sequencing, and species identification methodology is given in S1 Appendix.

### 3. Trace elements assessment

Methodology for trace elements analysis was based on previous work by [6]. Analyses of 14 trace elements (Ag, As, Cd, Co, Cr, Cu, Fe, Mn, Ni, Pb, Se, Sn, V, Zn) were realized with a Varian Vista-Pro ICP-OES and a Thermofisher Scientific XSeries 2 ICP-MS. Details on the methodology can be found in S1 Appendix.

### 4. Organic contaminants assessment

For 5 to 10 scallops per site, and for each of the two sampling periods, 21 to 23 PAHs, 14 PCBs and 8 pesticide residues were analyzed in digestive glands by stir bar sorptive extraction-thermal desorption-gas chromatography-tandem mass spectrometry (SBSE-GC-MS/MS) using a method adapted from [21]. Details on the methodology can be found in S1 Appendix.

### 5. Biomarkers assessment

Protein assays and the methodology used for each biomarker are described in S1 Appendix.

#### 5.1. Malondialdehyde (MDA) assay

Oxidative stress was evaluated through lipid peroxidation by quantifying malondialdehyde (MDA) that is a chemical metabolite of cell lipid degradation. MDA concentration was estimated in the final fraction using a commercially available MDA assay kit (Oxis International).

#### 5.2. Superoxyde Dismutase (SOD) assay

Superoxide dismutase (SOD) activity was measured given its important role in antioxidant response [22]. SOD activity was assessed in the final fraction using the method developed by [23].

#### 5.3. Laccase assay

Immune system alteration was evaluated by assessing a phenoloxidase (PO) activity (laccase-type) since this enzymatic activity has been shown to be contaminant-sensitive [24,25] and to play an important role in the immune defense mechanism in invertebrates.

#### 5.4. Glutathion-S transferase (GST) assay

Glutathion S-transferase (GST) activity was measured given its important role in contaminant excretions. Total activity of GST (GST are a group of enzymes) was determined according to the method of Sigma-kit (CS0410-1KT).

#### 5.5. Citrate Synthase (CS) assay

Citrate synthase activity has been extensively used as a metabolic marker in assessing oxidative, mitochondrial abundance and respiratory capacity [26,27]. CS activity was determined spectrophotometrically according to the method of [28].

#### 5.6. Evaluation of phosphatase activities

As phosphatases are involved in basic biochemical processes in marine invertebrates [29], we screened their activity in extracts of digestive glands. Phosphatases are involved in many lytic processes associated with the work of lysosomes and underlying anabolism.

### 6. Statistical analyses

Results are expressed as means ± one standard error unless otherwise specified. Statistical analyses were carried out using R v. 3.1.2 and 3.2.2 [30]. For all variables, normality was first tested on residuals using Kolmogorov-Smirnov tests and homogeneity of variances was assessed using Bartlett tests. For inorganic and organic contaminants and for phosphatase activity, values from three presumably more contaminated sites were compared to values obtained for the reference site (Loix-en-Ré) using One-Sample t-tests. For biomarkers, we conducted analyses of variance (One-way and Two-way ANOVAs) to highlight significant differences among sampling areas, sampling seasons, and interactions between these factors. If a significant difference was detected (p < 0.05), pairwise comparisons among conditions were conducted using a HSD Tukey post-hoc test.

We explored the multivariate response of biomarkers for our four sampling sites using Canonical Correspondance Analysis (CCA), in which ordination is constrained by environmental data, represented here by trace metal concentration in tissues. Stepwise model selection based on an AIC-like statistic was performed to determine which combination of trace metals optimized the fit of the CCA ordination. Individuals with missing data (n = 11) were removed from the analysis. CCA and its statistical significance (estimated using an ANOVA-like procedure based on 999 permutations) as well as model selection were performed in R using package vegan (functions cca, step and anova.cca; [31]).

### 7. Genetic diversity assessment

To estimate genetic diversity, *cox1* sequences were aligned by eye in Se-Al v. 2.0a11 [32]. Two shorter *cox1* sequences (from Les Minimes), which were used for species identification, were not included in the final alignment to estimate genetic diversity. The number of haplotypes (*H*), haplotype diversity (*Hd*), and nucleotide diversity (π) were calculated using DnaSP v. 5.10 [33], for each sampling site separately and excluding sequence sites with missing data. Data from the two sampling seasons were pooled. A median-joining network [34] was constructed to visualize relationships among haplotypes using PopART (available at: http://popart.otago.ac.nz) with default settings. A one-sample t-test comparing nucleotide diversity in the reference site with nucleotide diversity observed in the three other sites was conducted in R v. 3.1.1. [30].

Interpreting patterns of genetic diversity requires information on population structure across the geographic range that is sampled. Therefore, we tested whether individuals from the four sampling sites belong to the same panmictic population by performing an Analysis of Molecular Variance (AMOVA) in Arlequin v. 3.5.1.2 [35]. An estimator of the fixation index (*F*_ST_) was calculated based on haplotype frequency differences only and statistical significance was assessed using 10,000 permutations with the null hypothesis that *F*_ST_ is not different from zero (*i*.*e*., there is no genetic structure).

## Results and Discussion

### 1. Species validation

All individuals but one (from Loix-en-Ré) successfully amplified, and good quality sequences spanning 461 to 538 bp of the gene were obtained for 118 individuals (Loix-en-Ré: 29; Minimes: 29; Les Palles: 30; Port Neuf: 30). Sequences generated for this study were deposited in Genbank (accession numbers: KU680872-83). Prior to this study, only four *cox1* sequences of variegated scallops were available in public databases. All 118 sequences obtained in this study were genetically closest (~98% sequence identity) to the variegated scallop (*M*. *varia*) sequences already in Genbank (accession numbers EU523665-66), validating species identifications made in the field. The importance of this taxonomic verification step has been acknowledged by several ecotoxicological studies, for organisms collected in the field (*e*.*g*., [36,37]), and for toxicity studies involving test species commonly cultured in laboratories (*e*.*g*., [38]). Taxonomic uncertainty can be due to cryptic species (*i*.*e*., distinct evolutionary lineages lacking obvious characters distinguishing them morphologically), which, if not recognized, can confound results as they may respond differently to contaminants (*e*.*g*., [39–41]). In the present study, the low number of pairwise nucleotide differences among sequences was not suggestive of cryptic lineages.

### 2. Trace elements concentrations

Trace element concentrations are presented in Table 2 for both sampling dates. First, we investigated differences in concentrations between sampling periods. Differences across seasons were apparent for six heavy metals: Ag, Fe, As, Cd, Cr and Zn. Indeed, scallops of Loix-en-Ré and Les Minimes showed a higher bioaccumulation in September than in March (19% and 43% respectively) for Ag. Similar results were observed for Fe for the same sites, while the opposite pattern was detected for As, Cd, Cr and Zn bioaccumulation at all sites. For instance, a decrease in As concentration (30% for Loix-en-Ré and 53% for Les Palles) and a decrease in Cd concentration (62% for Les Minimes) were recorded.

**Table 2:** Trace element concentrations (μg/g of dry weight) in the digestive glands of *Mimachlamys varia* (7<n<10 per sites) from the four sampling areas (Loix-en-Ré, Minimes, Port-Neuf, Les Palles) in March 2014 and in September 2014.

March	LOQ (μg/kg of dry weight)	Loix	Minimes	Port-Neuf	Les Palles
**Ag**	0.06	7.67 ± 3.58	9.28 ± 0.88**	8.86 ± 0.66	12.67 ± 1.33***
**As**	0.75	17.49 ± 1.79	14.27 ± 0.44***	15.12 ± 0.40*	18.20 ± 0.67
**Cd**	3.72	25.02 ± 7.70	81.65 ± 6.22***	42.41 ± 4.01**	42.97 ± 3.22**
**Co**	0.06	0.69 ± 0.08	0.97± 0.07*	0.96 ± 0.05*	0.89 ± 0.03*
**Cr**	0.06	1.15 ± 0.35	2.25 ± 0.17*	2.57 ± 0.35***	2.09 ± 0.22*
**Cu**	3.72	33.04 ± 15.21	166.37 ± 30.24***	63.68 ± 6.13**	38.43 ± 6.66
**Fe**	14.91	443.38 ± 162.30	566.87 ± 82.44	908.04 ± 125.68***	893.76 ± 75.65***
**Mn**	3.72	17.76 ± 3.99	18.49 ± 3.69	28.18 ± 2.97*	25.50 ± 1.91*
**Ni**	1.5	2.79 ± 2.48	2.62 ± 0.28	2.76 ± 0.31	3.23 ± 0.36*
**Pb**	0.06	5.13 ± 2.41	6.39 ± 0.86	9.05 ± 1.27***	5.54 ± 0.59
**Se**	1.08	10.27 ± 1.64	11.91 ± 0.48	12.17 ± 0.33	16.17 ± 0.51***
**V**	4.5	2.62 ± 0.82	1.69 ± 0.32*	2.85 ± 0.36	2.24 ± 0.33
**Zn**	14.91	67.63 ± 7.12	112.98 ± 14.86***	119.81 ± 13.67***	221.55 ± 42.25***
**Sn**	0.06	< LOQ	< LOQ	< LOQ	< LOQ
**September**					
**Ag**	0.06	9.48 ± 1.52	16.29 ± 1.88**	13.48 ± 2.14*	11.19 ± 2.4
**As**	0.75	12.24 ± 1.15	9.17 ± 0.49	9.07 ± 0.47	8.45 ±0.38**
**Cd**	3.72	21.51 ± 2.83	30.73 ± 2.56	32.09 ± 3.19**	20.81 ± 2.22
**Co**	0.06	0.73 ± 0.06	0.58 ± 0.03**	0.51 ± 0.03	0.37 ± 0.01***
**Cr**	0.06	0.57 ± 0.07	0.90 ± 0.08**	0.50 ± 0.06	0.54 ± 0.05
**Cu**	3.72	31.73 ± 4.68	112.36 ± 21.48***	66.55 ± 7.5*	19.60 ± 2.27
**Fe**	14.91	612.35 ± 58.38	827.65 ± 92.17*	480.04 ± 40.26	729.06 ± 53.11
**Mn**	3.72	12.70 ± 0.84	24.44 ± 2.21***	14.93 1.41	20.78 ± 2.28
**Ni**	1.5	1.42 ± 0.13	1.11 ± 0.07***	1.06 ± 0.09	0.64 ± 0.03
**Pb**	0.06	0.99 ± 0.06	1.15 ± 0.09	0.92 ± 0.06	1.17 ± 0.05
**Se**	1.08	6.24 ± 0.53	5.06 ± 0.28	5.55 ± 0.27	5.52 ± 0.17
**V**	4.5	3.07 ± 0.29	2.47 ± 0.18	1.97 ± 0.16	2.76 ± 0.07
**Zn**	14.91	63.24 ± 3.74	62.08 ± 3.87	61.13 ± 4.44	65.13 ± 7.16
**Sn**	0.06	NA	0.06	NA	0.03

Stars (*, **, ***) above values indicate a significant difference in comparison with values obtained for the reference site (Loix-en-Ré), where p < 0.05, p < 0.01 or p < 0.001 respectively, LOQ is Limit Of Quantification.

Second, we compared trace element concentrations among sites, for each sampling season (March and September). In March (Table 2), Ag concentrations were significantly lower in Loix-en-Ré (reference site) than at two other sites: Les Minimes (sailing harbour) and Les Palles (near the mouth of the Charente Estuary). Concentration of seven metals (Ag, Cd, Co, Cr, Cu, and Zn) was significantly higher in scallops from Les Minimes, than in scallops from Loix-en-Ré. Higher concentrations for the three impacted sites compared with the reference site was observed for four metals: Cd, Co, Cr and Zn. Overall, metal concentrations we measured in March in Loix-en-Ré were similar to concentrations reported by [6] at the same sampling site.

In September (Table 2), significant differences were less pronounced. Indeed, only Ag, Co and Cu concentrations were significantly different for two impacted sites compared with Loix-en-Ré. Concentrations of traces elements Cd, Cr, Fe and Mn showed higher values either for Les Minimes or for Port-Neuf than in the reference site.

Many studies conducted on the bioaccumulation capacity of pectinids have been done in controlled condition of contamination [42], while our study was carried out using scallops collected *in situ*. Our results showed high bioaccumulation of Cd in the digestive gland of *M*. *varia* (25 to 81 μg/g of dry weight for March), which is consistent with previous findings on pectinids [6,14]. Previous studies indicated high levels of Cd in digestive glands in contaminated areas, as well as in reference sites [11,42–44].

Even though significant differences between the reference site and contaminated sites were not detected for all trace metals, the overall pattern that was observed did show that in Winter, most of these metals (11 out of the 14 analyzed) were present in lower concentrations at the reference site compared to one or several of the other sites. For trace elements showing higher levels in reference sites (e.g., for As), results could be explained by differences in dietary availability (e.g., [45]). In general, pectinids are filter feeders using different kinds of nutrients (phytoplankton, organic material and other suspended matter) with variable composition of nitrogen and carbon [46]. Thus, differences in diet among sampling areas could also explain the different metal contents. Additionally, previous studies have highlighted an influence of biotic and abiotic factors on metal concentrations in pectinids especially with seasons: Fall and Winter are marked by high concentrations. These variations can be due to temperature, nutritional intake and sexual cycle [42].

Bioaccumulation capacities of inorganic contaminants in bivalve mollusk organisms depend on tissue characteristics in target organs: kidney, digestive gland. Our study focused on the digestive gland, however it is important to underline an important bioaccumulation in the kidney of pectinids, mostly for essential metals [11,42–44]. Furthermore, it is difficult to infer accumulation pathways of metals from measures in one organ. Given the role of the digestive gland in digestion, the trophic pathway may be the main pathway of accumulation for this organ. However, seawater could also play an important role in global bioaccumulation of heavy metals. An organotropism study would shed some lights on these questions but interpretations should be made with caution [14].

### 3. Organic contaminants

A selection of 11 organic contaminants (pesticides, hydrocarbons and PCB) measured in the digestive glands of *M*. *varia* is presented in Table 3 (March and September 2014): two pesticides (aldrin and dieldrin), two PCBs (PCB 153 and 180) and seven PAHs (pyrene, fluoranthene, naphthalene, acenaphthylene, acenaphthene, fluorene and anthracene). Additional compounds (six pesticides, 12 PCBs and 15 to 16 PAHs) were measured (S1 Table).

**Table 3:** Organic contaminants concentrations (μg/kg of dry weight) in the digestive glands of *Mimachlamys varia* (5<n<10 per sites) from the four sampling areas (Loix-en-Ré, Minimes, Port-Neuf, Les Palles) in March 2014 and in September 2014.

March	LOQ (μg/kg of dry weight)	Loix	Minimes	Port-Neuf	Les Palles
Aldrin	1	2.24 ± 0.11	2.71 ± 0.54	ND	< LOD or LOQ
Dieldrin	1	6.38 ± 0.35	12.42 ± 2.79*	ND	4.15 ± 0.40
PCB 153	1	16.33 ± 2.07	78.09 ± 23.48**	ND	17.55 ± 4.20
PCB 180	1	3.34 ± 0.29	10.30 ± 2.71*	ND	3.49 ± 0.64
Pyrene	1.1	69.51± 12.78	127.74 ± 10.67*	30.54 ± 7.29	241.96 ± 121.84**
Fluoranthene	2	92.72 ± 15.01	190.02 ± 22.87*	40.70 ± 9.12	337.65 ± 183**
Naphtalene	2.9	32.41 ± 3.30	33.16 ± 3.36	90.01± 42.14**	34.81 ± 4.06
Acenaphtylene	0.9	3.18 ± 0.22	5.57 ± 1.12	2.41 ± 0.48	2.80 ± 0.83
Acenaphtene	1	4.12 ± 0.6	7.83 ± 1.49	2.29 ± 0.35	23.23 ± 11. 43**
Fluorene	0.3	3.76 ± 0.32	6.49 ± 1.48	6.73 ± 1.23	46.31
Anthracene	0;6	6.86 ± 1.14	18.61 ± 2.23*	4.44 ± 0.77	35.52 ± 21.31**
**September**					
Aldrin	14	<LOD	2.71 ± 0.54	<LOD	<LOD
Dieldrin	2,8	3.15 ± 0.34	7.08 ± 1.07**	5.71 ± 0.56*	4.50 ± 0.49
PCB 153	2,8	33.87 ± 4.86	77.37 ± 9.07***	84.91 ± 8.92***	52.91 ± 4.66*
PCB 180	14	< LOD or LOQ	< LOD or LOQ	12.78 ± 0.34	<LOQ
Pyrene	2,8	26.38 ± 3.42	38.28 ± 3.77*	30.54 ± 7.29	85. 08 ± 20***
Fluoranthene	28	52.03 ± 5.24	75.11 ± 11.29*	64.20 ± 4.78	135.83 ± 30***
Naphtalene	28	44.57 ± 3.57	35.04 ± 4.56	33.28 ± 3.57	37.92 ± 3.48
Acenaphtylene	14	<LOQ	<LOQ	15.90 ± 0.04	13.26 ± 0.57
Acenaphtene	28	<LOQ	<LOQ	<LOD	< LOD or LOQ
Fluorene	28	< LOD or LOQ	<LOD	< LOD or LOQ	< LOD or LOQ
Anthracene	14	7.78 ± 0.43	10.71 ± 1.18*	<LOQ	14.88 ± 2.27***

Stars (*, **, ***) above values indicate a significant difference in comparison with values obtained for the reference site (Loix-en-Ré), where p < 0.05, p < 0.01 or p < 0.001 respectively, and LOQ indicates Limit of Quantification (μg/kg of dry weight). ND: not detected

The comparison of the two sampling periods shows that lower organic contaminants rates were found in September 2014 than in March 2014, in digestive gland of scallops. Indeed, it was possible to note in Loix-en-Ré (reference site) a decrease of 50%, 62% and 43% of the bioaccumulation in digestive gland of dieldrin, pyrene and fluoranthene compounds respectively. These results are in accordance with previous studies performed on the Atlantic coast. Indeed significant differences of PAH level accumulated in bivalves were found between Summer and Winter, with maximal levels in Winter, both in oysters and mussels collected in the Bay of Biscay [47] and for juvenile oysters transplanted for a period of three months in different sites on the Marennes-Oléron Bay and the Gironde estuary [5].

Concerning pesticides, dieldrin showed a significantly higher concentration in digestive gland of scallops from Les Minimes, near sailing harbor, compared to Loix-en-Ré, both in March and September, and also in Port-Neuf in September. DDE is a degradation product of DDT, whose sell is prohibited since 1972 in France. Nevertheless, both compounds are ubiquitous, as they are very persistent in the environment [48]. 4.-4.DDE was found at the same level in Loix-en-Ré and Les Palles (see S1 Table), although inputs from agriculture are more important in Les Palles situated near the estuary of the Charente River. This is in accordance with levels measured in oysters by the French monitoring network ROCCH (Réseau d’Observation de la Contamination Chimique du littoral), who showed equal levels for DDT and its metabolites in five different sampling areas on the Marennes-Oléron Bay and the Gironde Estuary [5]. These organochlorine pesticides were transported along the entire coast and can now be found in equal quantities in open areas, making it impossible to find a reference site for pesticides. It has to be noted that 4.-4.DDE was found in higher quantity in scallops from Les Minimes, which constitutes a semi-open area, in which pollutants may concentrate.

Concerning PCBs, PCB 180 presented a significantly higher rate in Les Minimes in March, which was not observed in September. PCB 153 showed significantly higher concentrations in digestive glands of scallops from Les Minimes compared to Loix-en-Ré in March, and in scallops from the three polluted sites (Les Minimes, Port-Neuf and Les Palles) in September. Although the use of PCB was restricted, leading to a decrease of PCB levels in recent years, values of PCB 153 found in 2014 in the digestive glands of scallops in Les Palles (17.55 and 52.91 μg/kg of dry weight) were higher than those measured by ROCCH at the same site in oysters from 2003 to 2007 (9 and 12 μg/kg of dry weight) [5] and than values found for mussels in Arcachon Bay in 1997 (8.75 μg/kg of dry weight) [49]. This is all the more significant since mussels showed the highest bioaccumulation factor for PCB compared to razor shell and carpet shell [49]. Our results would indicate that scallops present a high bioaccumulation potential, compared with other bivalves. This was already pointed out for metal pollutants [12].

Regarding PAHs, Les Palles was the sampling site with the most contaminated scallops, with significantly higher concentrations in pyrene, fluoranthene, acenaphtene and anthracene compared to Loix-en-Ré, both in March (p < 0.01) and in September (p < 0.001). Pyrene, fluoranthene and anthracene were also measured in significantly higher concentration (p < 0.05) in scallops from Les Minimes in both sampling periods. These two contaminated sites (Les Palles, opposite to Oléron Island and Les Minimes, near the harbour of La Rochelle) are impacted by tourism practices during September and shipping throughout the year. Finally, naphtalene was found in significantly higher concentration in scallops from Port-Neuf compared to Loix-en Ré. Pyrene and fluoranthene have the highest rates among the accumulated PAHs accumulated in digestive glands of scallops. Both of these molecules contain four aromatic rings, which classifies them as heavy PAHs. Other heavy PAHs (benzoanthracene, benzo(b)fluoranthene, benzo(k)fluoranthene, benzo(a)pyrene, benzo(e)pyrene, perylene) were found in high levels in a least one sampling site (see S1 Table). These data are in agreement with previous results showing that other mollusks collected in the Atlantic coast, like oysters and mussels have a strong ability to accumulate more hydrophobic high molecular weight PAHs [5,47]. Fluoranthene levels measured in Les Palles in the present study (135.83 ± 30 μg/kg of dry weight in September and 337.65 ± 183 μg/kg of dry weight in March) are much higher than those obtained by ROCCH between 2003 and 2007 in the same sampling site for adult oysters collected *in situ* (21 ± 0 μg/kg of dry weight in Summer and 4±2 μg/kg of dry weight in Winter [5]). This could be due to the fact that ROCCH uses whole soft part of oyster, whereas digestive glands are used in the present study and PAHs have been shown to best store up in hepatopancreas in invertebrates [50]. Furthermore, *M*. *varia* was found to bioaccumulate more PAHs than many other bivalves found in Charente-Maritime, including oysters (*Crassostrea gigas*) and mussels (*Mytilus edulis*) [51]. Some of the PAHs we measured in September in the digestive glands in *M*. *varia* are also much higher than those measured in the digestive gland of *M*.*edulis* at the same period of the year, in the commercial port of Brest (Brittany, France), which has a severe record of chemical contamination with concentrations of PAHs (for example, Naphtalene 2.2 ± 0.3 μg/kg of dry weight in Brest and 33.28 ± 3.57 μg/kg of dry weight in Les Minimes; Fluoranthene 14 ± 2 μg/kg of dry weight in Brest and 75.11 ± 11.29 μg/kg of dry weight in Les Minimes) [52].

Organic contaminants measurements in the digestive gland of *M*. *varia* have provided information about anthropogenic sewage in four separate sites. In the present work, the data we obtained have shown high levels of dieldrin, hydrocarbons and PCBs (153 and 180) in Port-Neuf (Table 3) with important differences in comparison with the reference site. This might be explained by the presence of the sewage treatment plant of La Rochelle. Recent research indicates that the major sources of PCBs came from domestic effluent and rainwater [53]. Taken together, these different rates confirmed that Port-Neuf remained highly contaminated, which is coherent with previous results about heavy metals [6,11,13].

The results shown in Table 3 indicate that the mean total of PCB concentrations in scallops in Les Minimes during March and September were significantly higher than in the reference site (Loix-en-Ré). Moreover, measurements of pyrene and other congeners in digestive glands showed environmental forcing and harbour activity. These results might be due to the proximity of the dredging spoils of La Rochelle harbour.

Concerning Les Palles near the mouth of the Charente Estuary, hydrocarbons measurement results (p < 0.01) are unexpected and surprisingly high in comparison with pesticides low rates. Pyrene and Fluoranthene concentrations could potentially originate from other sectors around the Charente Estuary (Oléron Island, la Rochelle) with tidal currents, and in particular from Gironde Estuary. Moreover, previous research indicates that pesticides, PCBs and PAHs concentrations in sediments are higher than in the surrounding water, and this might have accounted for the observation in digestive glands of *M*. *varia*, which is a benthic species.

### 4. Biomarkers

The two-way ANOVAs indicated that the overall level of response differed between the two sampling seasons for all biomarkers (p < 0.006) except citrate synthase (F_1,69_ = 0.129, p = 0.7). Additionally, there was a significant interaction between the date and site of sampling for three biomarkers: laccase (F_3,68_ = 15, p < 0.001), malondialdehyde (F_3,70_ = 2.8, p = 0.045) and superoxyde dismutase (F_3,66_ = 13, p < 0.001), which signifies that differences among sites were not the same for the two seasons. Seasonal variation in biomarker levels may be due to the reproductive status of variegated scallops at time of sampling, as well as abiotic factors. Indeed, September sampling was conducted at the end of these organisms’ reproductive period, when perhaps less energy can be allocated to defense mechanisms.

In some datasets (e.g., Laccase dataset, for which variance at Les Minimes was higher than at other sites), residuals deviated from normality and variances were non homogeneous. Results from one-way ANOVAs were therefore checked using Kruskall-Wallis tests, which provided the same results as the ANOVAs.

Except for malondialdehyde, all biomarker levels differed among sites in March (p < 0.03), while three biomarkers (Laccase, malondialdehyde and superoxyde dismutase) showed significant differences among sites in September (p < 0.004). Results for each biomarker is further presented and discussed below.

#### 4.1. Laccase assay

**Fig 4:**
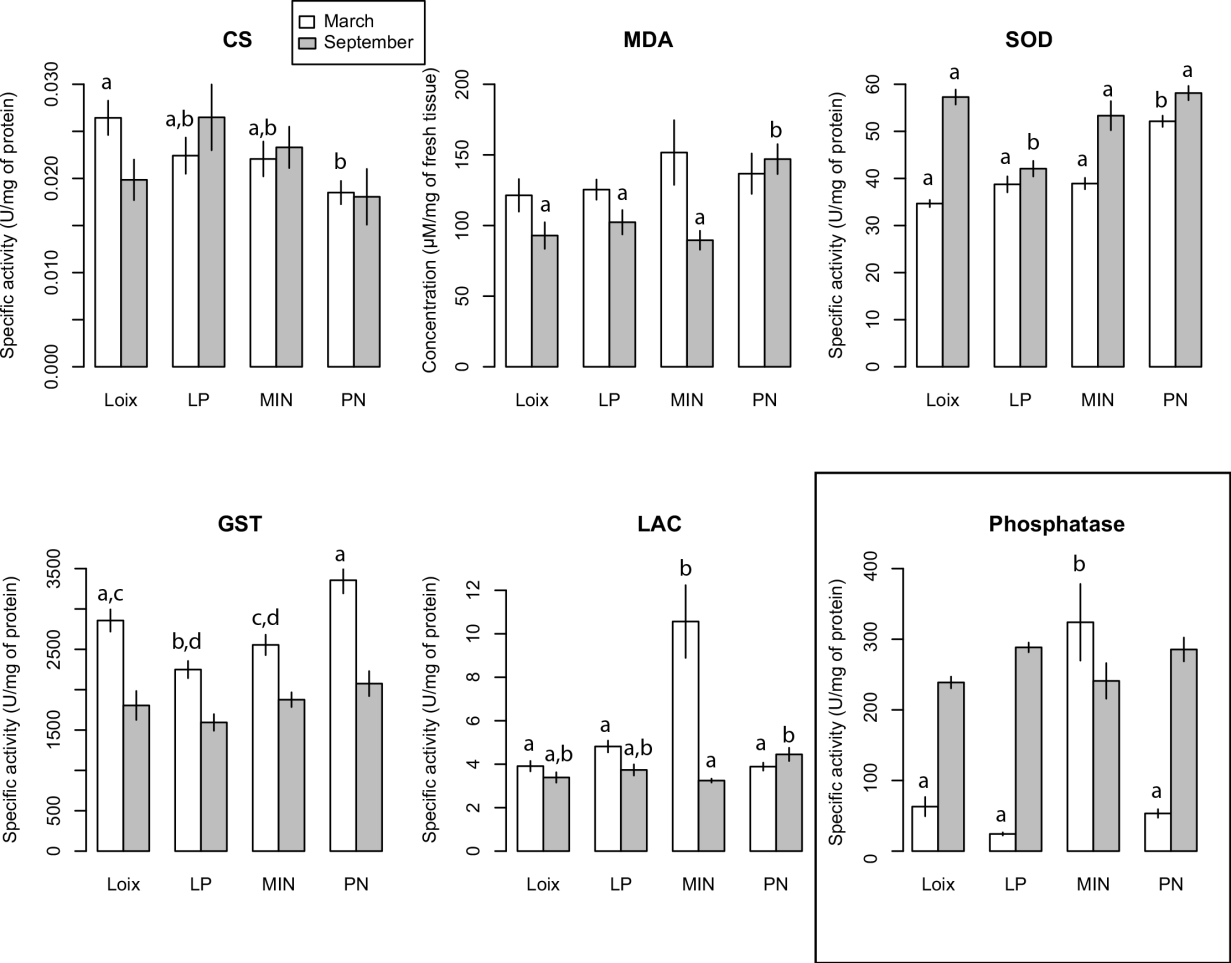
Biomarkers and phosphatase activity assessed in the final fraction of *Mimachlamys varia* digestive glands or muscle (see Material and Methods) for March and September 2014 from Loix-en-Ré (Loix), Les Minimes (MIN), Port-Neuf (PN) and Les Palles (LP). CS: Citrate synthase activity, MDA: MDA contents, SOD: SOD activity, GST: GST activity, LAC: Laccase activity. Note that the barplot for phosphatase activity is separated by a frame as this activity was measured using 10 pooled individuals (see Material and Methods). Sampling seasons (March and September) are color-coded and are shown on the same barplot. Values represent mean ± standard error (n = 10 individuals per site for biomarkers; n = 3 replicate measurements of the pool for Phosphatase activity). Within each season, bars that do not differ significantly (p > 0.05) share the same letters. No letters are shown above bars when there was no significant statistical difference among sites.

In March, Laccase activity was 10.6 ± 1.7 U/mg of protein for organisms sampled in Les Minimes (Fig 4). When compared to this value, the three other sites presented significantly lower levels of activity for this enzyme. Conversely, Port-Neuf showed a significantly elevated activity compared to Les Minimes in September (Fig 4).

The values obtained are in agreement with previous works in other bivalve species *Crassostrea gigas* and in scallop *M*. *varia* [6,54]. Laccase proves to be a sensitive biomarker and phenoloxydase seems to play an important role in bivalve immunity when they are activated by ROS [25]. Moreover Laccase has metal coenzymes that can be affected by heavy metals according to the same activation as SOD in short-time (part 3.4.2).

#### 4.2. Superoxyde Dismutase (SOD) assay

The mean value of SOD activity for March was 34.67 ± 0.89 U/mg in Loix-en-Ré (Fig 4). For March, scallops from Port-Neuf showed a significantly higher level of SOD activity compared with the three other sites. For the September sampling, a mean value of 55.39 ± 1.80 U/mg of protein (an increase of 37% in comparison with March) was measured in Loix-en-Ré. The activity of the enzyme significantly differed between Les Palles and the three other sites, the latter showing higher activity levels (Fig 4). Overall, mean SOD activity was significantly higher in September compared to March.

The increase in SOD activity observed in Port-Neuf (near a water treatment plant) in March may be linked to the important role of this enzyme in inhibiting ROS production and thus, decreasing lipid peroxydation effects in cells. This finding is in accordance with different studies conducted in pectinids that showed a modulation of this enzyme activity due to inorganic contaminants [6,55] and organic contaminants [56]. This higher activity may constitute an antioxidant response (probably induced by ROS) in *M*. *varia*.

#### 4.3. Malondialdehyde (MDA) assay

For the first date (March), MDA content in Loix-en-Ré scallops showed an average value of 119.50 ± 13.27 μM (Fig 4). No significant difference in MDA content was observed among the four sites for this season.

For the second sampling (Fig 4), lipid peroxidation showed a significant difference between Port-Neuf near water treatment of La Rochelle city and the three other sites. Indeed, MDA content in Port-Neuf scallops showed an average value of 147.0 ± 10.6 μM, representing a 63% increase compared to scallops from Loix-en-Ré (reference site). This result highlighted an increased lipid peroxidation in the scallops from one of the contaminated sites. This is consistent with the results of [55] who also showed this phenomenon studying *Chlamys farreri* from less contaminated and contaminated sites. These results suggest that MDA in Port-Neuf, assessed in the digestive gland of *M*. *varia*, is a biomarker responding to accumulation of peroxydation product, membrane destabilization which have been related with decrease of capacities of oxidative defenses [57].

#### 4.4. Glutathion-S transferase (GST) assay

The mean GST activity for first sampling in the digestive gland of *M*. *varia* was 2546.02 ± 139.45 U/mg of protein in Loix-en-Ré scallops (Fig 4). Compared to this value, scallops from Les Palles near the Charente estuary showed a significantly lower level of GST activity. Furthermore, there is a significant positive difference in this enzyme’s activity between Port-Neuf and two other sites (Les Palles and Les Minimes) (Fig 4).

In September (Fig 4), GST activities did not show significant differences among sites. Overall, GST showed a significantly lower activity at all sites in September in comparison with March. It is important to notice that this enzyme activity showed low intra-group variability. The seasonal difference we observed for this enzyme may result from the organism’s sexual reproduction cycle and abiotic factors.

A clear modulation of glutathione metabolism was mesured in mollusks collected at Loix-en-Ré, Port-Neuf, Les Minimes and Les Palles. Glutathion S-transferase (GST) plays an important role within the cellular oxidant system and in particular with the regulation pathway for detoxication of xenobiotics. A reduction in GST activity in September in digestive glands of scallops could be due to depletion levels of glutathione with an inhibition of glutathione reductase causing an imbalance of Warbour-Dickens-Horecker pathway (oxidative equilibrium). The depletion of this enzyme activity renders *M*. *varia* more susceptible to formed peroxides as also confirmed by the significant increase of malondialdehyde in Port-Neuf (see part 3.4.3).

#### 4.5. Citrate synthase (CS) assay

CS is one of the key regulatory enzymes in the energy-generating metabolic pathway that catalyzes the condensation of oxaloacetate and acetyl coenzyme A to form citrate in the citric acid cycle [58]. Variable changes in CS activity indicated the possible alterations in oxidative capacity [59]. The CS activity for both sampling seasons in the muscles of *M*. *varia* is presented in Fig 4. In Loix-en-Ré, mean value was 0.024 ± 0.003 U/mg of protein in March. The activity of the enzyme did significantly decrease for the Port-Neuf site near waste-water compared to the reference site of Loix-en-Ré. However, scallops from all contaminated sites, that is, Les Minimes, Port-Neuf and Les Palles showed 12.5%, 20.0%, and 17% significantly lower specific activities when compared to Loix-en-Ré, respectively (Fig 4).

For the second sampling in September (Fig 4), there was no significant difference among sites. High variation in glycogen levels during storage and gametogenic development suggests that carbohydrates are the main respiratory substrate [60]. Outside this metabolically active period, bivalves are probably less sensitive to stress.

Responses to environmental stress (temperature, salinity, pollutants…) in marine organisms induce changes in energy metabolism and oxidative stress [61,62]. In these reviews, Tomanek suggests that metabolic pathways are activated to respond to increased oxydative stress or to switch metabolic fuels. The ability to control metabolic processes in response to changes is indispensable. This adaptability is necessary for conserving the stability of the intracellular environment (homeostasis), and essential for maintaining an efficient functional state. Metabolic control is accomplished by altering the activity of at least one pacemaker enzyme (or rate-determining step) of the pathway. In our study, the decrease in CS activity at impacted sites suggests a limitation of the Krebs cycle to the benefit of another pathway. CS stands as a pace-making enzyme in the first step of this metabolic pathway.

#### 4.6. Phosphatase activity

Screening of phosphatase activity in the digestive gland of *M*. *varia* was conducted for the two seasons and for the four sampling areas. In the digestive system of these animals, the specific activity of phosphatase was 5–13 times higher in Les Minimes (0.324 μmol/mL/min/μg) than in the other sampling sites in March (Fig 4; one sample t-test: t_2_ = -23,8, p = 0.002). In September, specific activity of phosphatase was higher than those measured in March (> 0.28 μmol/mL/min/μg for all sites) but we found no difference among sampling sites. Similarly to laccase activity, a high value of specific activity was recorded in March and in Les Minimes corresponding to a pre-reproductive period for animals. Phosphatases are known to be implicated in regulation of metabolic pathways such as gluconeogenesis and glycogenesis, and are sensitive to heavy metals [63]. Therefore, we suggest that *in situ* contamination of Les Minimes by heavy metals like Cd may modify the capacity of scallops to produce glucose and glycogen, the primary carbohydrate stored in the liver and muscle cells of animals. Overall, variations in both phosphatase and citrate synthase activities suggested metabolic changes in *M*. *varia*.

### 5. Relationship among biomarker responses and contaminants

**Fig 5:**
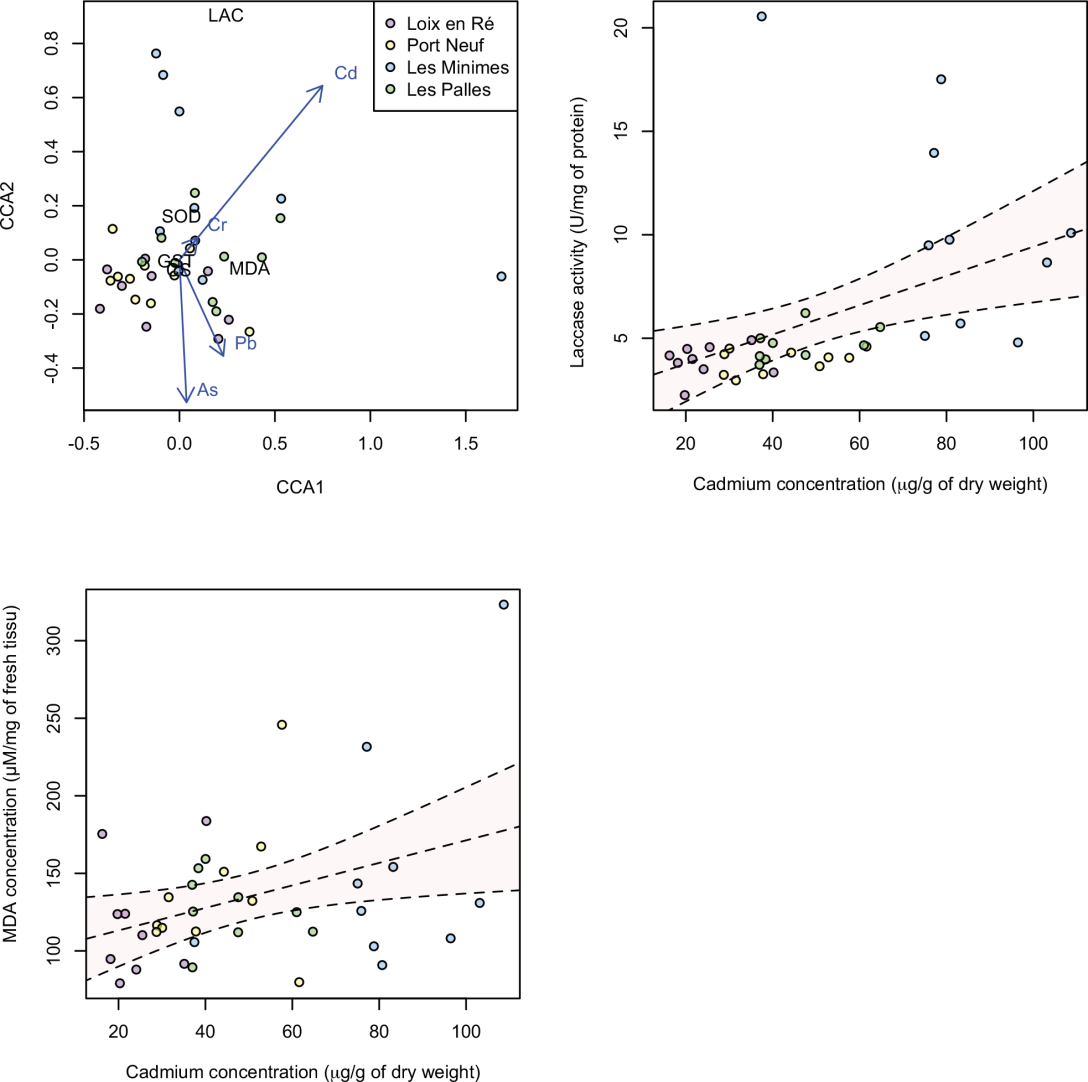
Canonical Correspondance Analysis (CCA) ordination plot of biomarker responses for individuals collected in March at four sampling sites (represented by different colors), and the trace metals explaining the variation in the individual x biomarker matrix (blue arrows). Biomarker values were scaled to ⅓ as to fit on the plot. The first two axes explain 44% of the variance in the data (40% and 4%, respectively). CS: Citrate synthase activity, MDA: MDA contents, SOD: SOD activity, GST: GST activity, LAC: Laccase activity.

CCA were run on each sampling season separately prior to attempting analyses pooling the two sampling dates. For the month of September, constrained analyses had a poor fit and were therefore not pursued, and consequently an analysis pooling both sampling seasons was not attempted. For the month of March, a model including Cd, As, Pb and Cr was selected. The ANOVA-like function of the R package vegan suggested that this constrained analysis was statistically significant (anova.cca function; 999 permutations, p < 0.001). This model, explaining 44% of the variance in the dataset (axis 1: 40%; axis 2: 4%), suggested that Cd was the most structuring factor of the environmental matrix (anova.cca permutation test: p < 0.001), and most particularly the variation in the Laccase and MDA biomarkers (Fig 5). Indeed, both Laccase and MDA levels were positively correlated with Cd concentrations in the tissues of *M*. *varia* (linear models: R^2^: 18%, F_1,36_ = 9.0, p = 0.005 for LAC and R^2^: 12%, F_1,36_ = 5.9, p = 0.021 for MDA), although variance in the MDA dataset was higher than in the Laccase dataset. Loix-en-Ré and Les Minimes displayed the lowest and the highest biomarkers and Cd concentrations, respectively.

Enzymatic activities of Laccase and MDA are involved in immune response and lipidic peroxidation, respectively. Thus, lipidic peroxidation, which acts on the plasma membranes by destabilizing fatty acid poly-insaturated, could be notably due to accumulation of Cd. Our results are in accordance with [6], which reported the induction of Laccase activity in harbour areas. An increase in Laccase activity reflects the synthesis of melanins from phenolic substrates, which plays a decisive role of cellular response to metal and in particular to Cd in *M*. *varia*.

Contrary to inorganic pollutants, it was impossible to explore the multivariate response of biomarkers using CCA with ordination constrained by organic pollutants concentrations in digestive glands. Indeed, whole digestive glands were used for organic contaminant measurements to maximize the sensitivity of the methodology, and biomarkers were assayed in other individuals. Therefore we can only highlight trends of simultaneous modulation of biomarkers and organic contaminants.

Our study of organic contaminants in digestive glands of *M*. *varia* showed, for the first time, a high bioaccumulation capacity of this marine organism for this type of contaminants. In parallel, significant changes were observed in activities of enzymes involved in the modulation of oxidative stress (GST, SOD), immune system alteration (Laccase), mitochondrial respiration (CS) and general metabolism (phosphatase) in scallops collected from impacted sites compared with less exposed scallops (data from March). This is in agreement with the existence of some chemical pressure in Les Minimes and Port-Neuf harbors and in Les Palles. This confirms previous results obtained in Marennes-Oleron Bay and Gironde Estuary, in juvenile oysters of *C*. *gigas* transplanted in contaminated areas. Indeed, higher levels for SOD, MDA, and Laccase were measured in winter in correlation with high levels of organic contaminants [5].

Regarding enzymes involved in the modulation of oxidative stress (GST, SOD), high GST and SOD activities were found in March in Les Minimes and Port-Neuf, where high levels of PAHs were found in the digestive glands. This suggests an enhancement of xenobiotic biotransformation processes and of antioxidant protection. This is in accordance with two studies conducted by [64,65] who showed an increase of GST activity in the digestive glands of pectinids contaminated with PAHs.

Laccase activity was high in Les Minimes in March, in comparison with the reference site (Loix-en-Ré). In addition to the potential response of Laccase activity to higher Cd concentrations mentioned above, this result could be linked to a modulation of enzymatic activity by organic contaminants (hydrocarbons and PCBs) [5,54].

A decline of citrate synthase (CS) activity and an increase in phosphatase activity in a contaminated site (Port-Neuf) compared with a reference site (Loix-en-Ré) was also found in March. These enzyme activity levels could be linked with high PAHs rates in the fishing harbor and other contaminants coming from the water treatment plant.

Our results showed potential links between modulations of biomarkers (SOD, MDA, GST, Laccase, CS) and chronic chemical contamination *in situ*. Although we identified trends between biomarkers and different classes of contaminants, it is important to note other parameters might have some effect on this biological response. Several abiotic factors such as oxygen level, temperature, pH and environment dilution could modulate enzymatic response in bivalves [66,67].

### 6. Genetic diversity assessment

**Fig 6:**
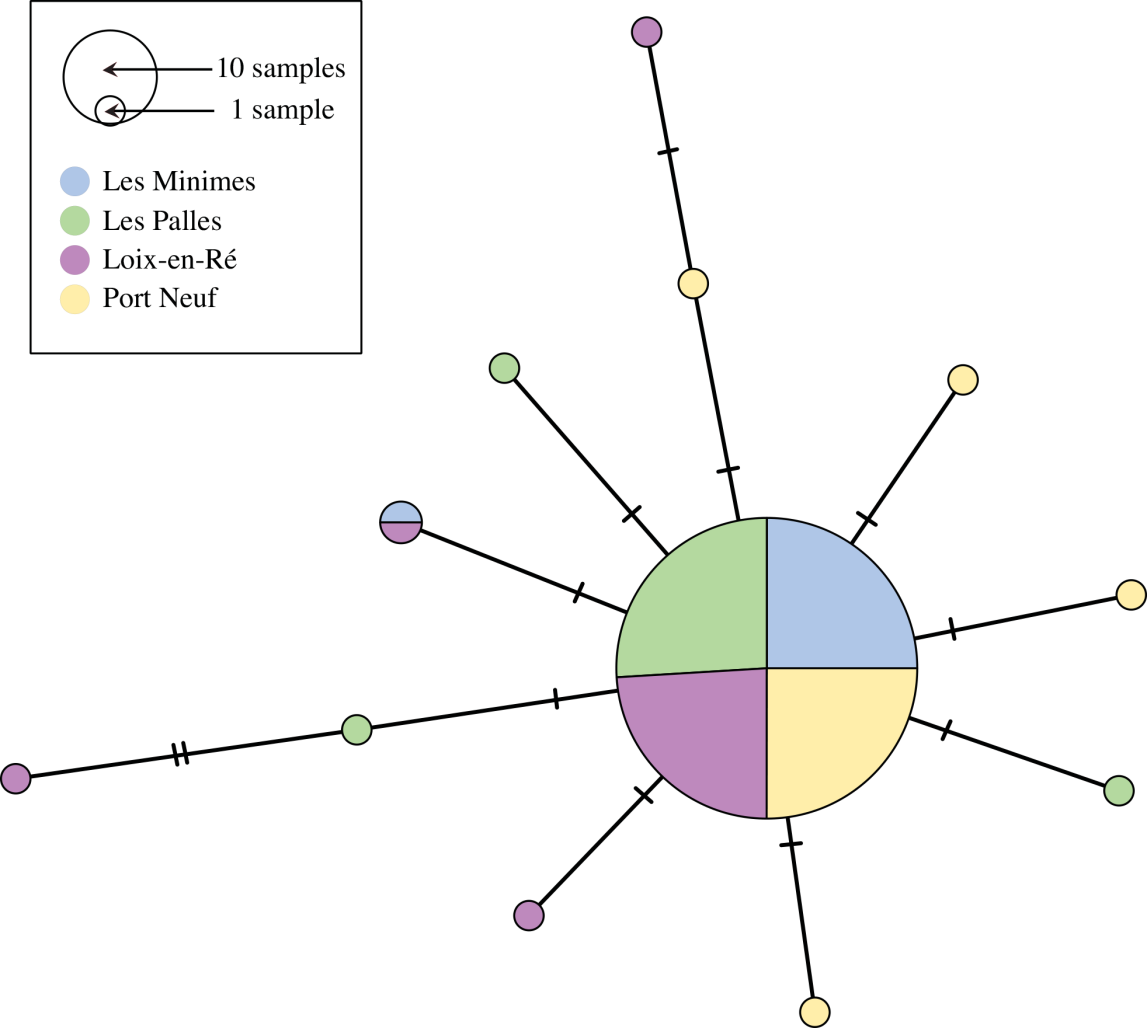
Median-joining network representing the 12 unique haplotypes found across 477 bp of the mitochondrial *cox1* gene in 116 *Mimachlamys varia* individuals from four sampling sites. Each filled circle represents a unique haplotype and its size is proportional to the number of individuals sharing this haplotype. Each circle is colored proportionally to the number of individuals from each sampling site. The number of mutations separating connected haplotypes is represented by tick marks on the line.

The final alignment used for genetic diversity assessments was 477 bp including seven sites with missing data that were excluded from analyses. The alignment included 12 variable sites that were all synonymous substitutions. A total of 12 haplotypes were found within the dataset, including one central haplotype that was the most common at all sites (Fig 6). The greatest genetic diversity was observed in Loix-en-Ré (the reference site for inorganic contaminants), particularly in terms of nucleotide diversity. Conversely, the lowest genetic diversity was measured in Les Minimes (Table 4). The difference in nucleotide diversity observed between the reference site (Loix-en-Ré) and the other three sites was statistically significant (one sample t-test: t_2_ = -5.3, p = 0.03).

**Table 4:** Genetic diversity indices for *Mimachlamys varia* (four sampling sites) calculated using sequences spanning 477 bp of the mitochondrial *cox1* gene. *N*: number of sequences analyzed; *H*: number of haplotypes; *Hd*: haplotype diversity; π: nucleotide diversity.

Sampling site	*N*	*H*	*Hd*	π
Loix-en-Ré	29	5	0.261	0.00103
Les Palles	27	4	0.193	0.00043
Les Minimes	30	2	0.074	0.00016
Port Neuf	30	5	0.253	0.00057

Measuring the consequences of chemical pollution on the genetic diversity of natural populations has been recognized as an important aspect in impact assessments [68]. However, only a limited number of ecotoxicological studies include a genetic component (but see for example: [69–71]). Chemical contaminants can affect genetic diversity by increasing the mutation rate, by causing a population size reduction (bottleneck), or through directional selection favoring some genotypes [72].

To properly interpret patterns of genetic diversity, we investigated genetic differentiation among sampling sites. The haplotype network (Fig 6) did not reveal any genetic structure. This was confirmed by results from the AMOVA: no significant restriction of gene flow was observed among the four sites (*F*_ST_ = -0.007 p = 0.7). Thus, all sampled individuals belong to the same population and differences in genetic diversity among sites are not due to genetic drift or differences in effective population size. Given the short distances observed among haplotypes (Fig 6), we can also rule out that the presence of cryptic species could be driving genetic diversity differences (see [73]). On the contrary, lower genetic diversity in more contaminated sites may result from the effect of selective pressure eliminating less resistant genotypes (a process that is plausible for mitochondrial coding genes) coupled with high larval mortality due to chemical pollution (which has been reported in marine bivalves: [74]). It is important to note that a direct causal relationship between contamination and genetic diversity cannot be established here, but the significant relationship we observed is consistent with findings from previous studies (*e*.*g*., [70,71,75,76]). Moreover, an implication of the same processes (high mortality and selection) has been suggested in other invertebrate models (*e*.*g*., [75,76]).

The consequences of our findings in terms of evolutionary potential and persistence of this population is unclear, particularly in a marine bivalve with presumably large effective population sizes. Nonetheless, a decrease in genetic diversity linked to anthropogenic impacts is generally seen as a negative population response, all the more if these impacts are expected to intensify in the near future.

Evaluating the effects of chemical pollution on gene expression levels in this model would be an interesting next step, particularly using a transcriptomic approach. The promise of ‘-omic’ methodologies for the study of immune response in marine bivalves has been recently highlighted by [77].

## Conclusions

Assessing the effects of chemical contamination on natural populations collected *in situ* is complex and requires the integration of i) multiple indicators of individual and population health and stress response, ii) different types of contaminants that may be present in the environment. Our integrative study applied to natural populations of variegated scallops underlines how different locations on the infralittoral zone can display significantly distinct contamination profiles despite being geographically close. By combining analysis of organic and inorganic pollutants, we showed that locations that are considered reference (i.e., less contaminated) sites for certain contaminants (here, trace elements), may be impacted by other types of contaminants. This finding has important implications for biomonitoring studies as the organisms’ responses will reflect the effects of the mixture of all contaminants present, referred to as cocktail effects.

Furthermore, including multiple sampling time points is crucial, as pointed out by the differences in contamination profiles and levels of biomarker responses we measured for March and September. These differences are likely due to the reproductive status of the organisms at time of sampling, which influences the energy that can be allocated to defense mechanisms during gametogenesis. Additionally, environmental conditions such as temperature, pH, oxygen levels and food availability may play a role in the seasonal differences we observed.

Our measurements of biomarkers and phosphatase activities revealed different physiological responses among sites, including the modulation of citrate synthase, a biomarker that was evaluated in *M*. *varia* for the first time. In terms of stress response, multivariate analyses of inorganic contaminants and biomarkers revealed a significant link between the activity of the laccase-type enzymes, and Cd contamination. Additionally, MDA content was also correlated Cd concentrations. Finally, we observed a significant decrease in genetic diversity at sites impacted by heavy metal pollution, suggesting long-term (intergenerational) effects of this chronic contamination at the population level.

Accumulation rates of contaminants in benthic organisms depends on various factors, such as exposure time, sediment nature, uptake and desorption by organisms and behaviour of bivalves during exposure. In contrast with the murine model (e.g. [78]), the fate of chemical organic contaminants in scallops has been poorly studied at this day. Given its bioaccumulation rates of both inorganic and organic contaminants, the variegated scallop, *M*. *varia*, appears as a good model for biomonitoring studies in the marine environment. Since *M*. *varia* is not farmed, it provides a better reflection of the natural environment than cultivated bivalves, and patterns of genetic diversity are more easily interpreted. Furthermore, monitoring contaminant levels in variegated scallops collected *in situ* is important for health and economic reasons as it is harvested for human consumption. Owing to the important bioaccumulation potential of the species, it will be interesting to elaborate a protocol for measuring both organic contaminants and biomarker responses on the same individuals, as it is already the case for inorganic contaminants. Our study provides novel baseline information on levels of organic contaminants and population genetic diversity for this bivalve on the way to becoming a sentinel species.

## Supporting Information

S1 Appendix Detailed methodologies for biochemical material preparation, genetic analysis, pollutant measurements and biomarker assessments. (DOCX)

S1 Table Supplementary results for organic contaminants concentrations. (DOCX)

S2 Table Individual data for biomarker levels and metal concentrations. (DOCX)

S3 Table Individual phosphatase activity data. (DOCX)
