# Supplementary material for: Short-Term and Long-Term Biological Effects of Chronic Chemical Contamination on Natural Populations of a Marine Bivalve
Source: PLoS One. 2016 Mar 3;11(3):e0150184. doi: 10.1371/journal.pone.0150184 (PMC4777565; doi:10.1371/journal.pone.0150184)
Supplement: S1 Appendix — (DOCX) [file pone.0150184.s001.docx]

**Breitwieser, Viricel *et al.* - Supporting Information**

**S1 Appendix**

**Detailed methodologies for biochemical material preparation, genetic analysis, pollutant measurements and biomarker assessments.**

*2.1 Preparation of biochemical material*

Digestive gland and adductor muscle were collected in the laboratory and stored at -80°C while awaiting analysis (Fig. 3), except for individuals used for phosphatase activity analysis for which digestive glands were dissected and immediately frozen in liquid nitrogen the day of sampling. Analyses of inorganic contaminants and of biomarkers were conducted on the same individuals:  the digestive gland was ground with a mortar and divided into two samples (one used for inorganic contaminant analysis, one for biomarker assessment).

For trace element analyses, samples were freeze-dried for 24 hours (CHRIST Beta 1-8 LD Plus).

For organic contaminants, assays were performed by the Centre de Documentation, de Recherche et d’Expérimentations sur les Pollutions Accidentelles des Eaux (Cedre, Brest, France) on digestive gland or whole tissue homogenate samples stored at -20°C until analyses. Biomarker assessment samples were homogenised in an ice-cold phosphate buffer (100 mM, pH 7.2, 1100 mOsm) with a protease inhibitor (halt protease inhibitor cocktail 87785, Thermo Scientific). The homogenates were centrifuged at 12500 g, at 4 °C for 15 min and the final fractions (FF) were used for biochemical assays.

For the citrate synthase assay, after weighting, muscle tissue was homogenized in ice-cold Tris-HCl Buffer (0.1 M Tris-HCl, 1 mM EDTA, 2% (v/v) Triton™ X-100, 7.4 pH : 1 mL for 80 mg tissue) using a pestle motor mixer (Argos Technologies Pellet Mixer). The homogenate was centrifuged (15 min, 10,000 g, 4°C). The supernatant containing the enzyme extract was collected and kept cold (4°C)  for the citrate synthase and protein assays.

2.2 *Species identification: genetic analysis*

Primers specific to the variegated scallop were designed using two *M. varia*, one *M. senatoria*, one *M. nobilis*, and one *Mizuhopecten yessoensis* sequences from Genbank (accession numbers: EU523665-66, NC022416, NC011608 and NC009081): forward primer Cox1F3 5’-GGGTTTGGRAACTGGCTTCT-3’ and reverse primer Cox1R-long 5’-CCAAAAACMCGAATCTTCTT-3’. The 35 μL reactions included 1X Reaction Buffer (biotechrabbit, Hennigsdorf, Germany), 1.5 mM MgCl_2_, 0.3 μM for each primer, 0.15 mM dNTPs, 1 U *Taq* polymerase (biotechrabbit, Hennigsdorf, Germany), and 9 to 38 ng DNA. The PCR profile was: 94°C for 2 min, followed by 30 cycles of 30 s at 94°C, 30 s at 54°C, and 30 s at 72°C and a final extension of 7 min at 72°C. No-template negative controls were included in DNA extractions and PCR reactions. PCR products were sent to Genoscreen (Lilles, France) for purification and Sanger sequencing in both directions. Sequences were edited manually using Sequencher^®^ v. 4.7 (Gene Codes Corp., Ann Arbor, MI, USA). Species identification based on *cox1* sequences was achieved in GenBank using the BLAST function with default parameters (<http://blast.ncbi.nlm.nih.gov/Blast.cgi>).

*2.3 Trace element assessment*

Each digestive gland sample was weighted (to the nearest 0.1g) and digested using a 3mL of 67–69% HNO_3_ and 1mL of 34–37% HCl mixture (Fisher Scientific, trace metal quality). Acidic digestion of the samples was carried out overnight at room temperature and then in a Milestone microwave oven (30 min with constantly increasing temperature up to 120°C, then 15 min at this maximal temperature). Each sample was adjusted to 25 mL with ultrapure quality water (Milli-Q). Two certified reference materials (CRMs) and one blank, treated and analyzed in the same way as the samples, were included in each analytical batch. CRMs were DOLT-4 (dogfish liver) and TORT-2 (lobster hepatopancreas) both from the National Research Council Canada (NRCC). Mean element concentrations are expressed in µg/g of dry weight (drying at 50°C). The detection limits were 0.02 (Ag), 0.25 (As), 1.24 (Cd), 0.02 (Co), 0.02(Cr), 1.24 (Cu), 4.97 (Fe), 1.24 (Mn), 0.05 (Ni), 0.02 (Pb), 0.12 (Se), 0.02 (Sn), 0.5 (V) and 4.97 (Zn) µg/g of dry weight.

*2.4 Organic contaminants*

For each organism, 100 mg wet weight (w.w.) of tissue were digested by saponification and analytes were extracted for 16 hours at 700 rpm using polydimethylsiloxane stir-bars (Twister 20 mm x 0.5 mm, Gerstel). Bars were subsequently analysed using a gas chromatography system Agilent 7890A coupled to an Agilent 7000 triple quadripole mass spectrometer (Agilent Technologies) and equipped with a Thermal Desorption Unit (TDU) combined with a Cooled Injection System (Gerstel). Thermodesorption and GC-MS/MS conditions were as previously described in Lacroix et al. (1). Analytes were quantified relatively to deuterated compounds using a calibration curve ranging from 0.1 ng to 30 ng per bar. A mean digestive glands water percentage of 64.3% was measured by drying samples at 50°C until mass remained constant. Results are expressed as µg analytes/kg dry weight (d.w.). Limits of quantification (LOQ) were calculated by the calibration curve method (2) and limit of detection (LOD) were estimated by dividing LOQ by 3. Analytical quality control was made using the Standard Reference Materials 1974c *“*Organics in Mussel Tissue *(Mytilus edulis)*” provided by the National Institute of Standards (SRM) and Technology (NIST, Gaithersburg, USA).

*2.5. Biomarker assessment*

*Protein assay*

For the Superoxyde Dismutase, Laccase and Glutathion-S transferase assays, protein concentrations in the final fraction (FF) were determined using Bradford method (3) with a BioRad assay kit (500-0002) that contained bovine serum albumin (BSA) as a standard and Brilliant Blue G 250 as a reactant. This reaction was measured at 570 nm using a spectrofluorometer (SAFAS Flx-Xenius).

For the Citrate Synthase assay, the solubilized protein muscular extracts were quantified in triplicate by using bicinchoninic acid reagents (BCA1 kit, Sigma) and bovine serum albumin standards.

*2.5.1. Malondialdehyde (MDA) assay*

The method was based on the reaction of MDA with a chromogenic reagent (n-methyl-2-phenylindole). The blue product was quantified by measuring absorbance at 586 nm (4) using the SAFAS Flx-Xenius spectrofluorometer. The results are presented in µM/ mg of fresh tissue.

*2.5.2. Superoxyde Dismutase (SOD) assay*

The assay, involving EDTA, MnCl_2_ and mercaptoethanol, measured the decrease of nicotinamide adenine dinucleotide (NADH) oxidation. This inhibition of oxidation was monitored spectrophotometrically at 340 nm (using the SAFAS Flx-Xenius spectrofluorometer) and is a function of SOD activity. Fifty percents of inhibition of oxidation correspond to one unit of SOD. The results were presented in enzyme unit of SOD per mg of proteins (U/mg of protein). Proteins were assayed by method of Bradford.

*2.5.3. Laccase assay*

Using a method developed by Luna-Acosta et al. (5), this activity was measured in the FF. The method is based on the fact that the oxidation of PPD (p-phenylenediamine) is catalysed by laccase. Thus, the activity of laccase-type phenoloxidase (PO) is a function of PPD degradation. The reaction was assessed at 420 nm using a SAFAS Flx-Xenius spectrofluorometer. In parallel, the non-enzymatic auto-oxidation (oxidation in the absence of FF) was subtracted. Laccase-type PO activity was expressed in enzyme unit per mg of proteins  (U/mg of protein with one unit is defined as the amount of enzyme that catalyses the appearance of 1 µmol of product per min). Proteins were assayed by method of Bradford.

*2.5.4. Glutathion-S transferase (GST) assay*

The Glutathione S-Transferase Assay kit utilizes 1-Chloro-2,4-dinitrobenzene (CDNB), which is suitable for the broadest range of GST isozymes. Upon conjugation of the thiol group of glutathione to the CDNB substrate, there is an increase in the absorbance at 340 nm. The GST activity was then normalized to the total protein content and was reported in enzyme unit per mg of proteins (U/mg proteins). Proteins were assayed by method of Bradford.

*2.5.5. Citrate Synthase (CS) assay*

CS activity in muscular extracts was determined spectrophotometrically according to the Srere method (6). Briefly, 5 µL of muscular extract were added in 185 µL of assay buffer (0.1 M Tris-HCl, 1mM EDTA, 0.112 mM DTNB, 0.33 mM AcétylCoA).  The reaction was started with the addition of 10 µL of oxaloacetate (10 mM). The activity was monitored at wavelength of 412 nm at 20 s intervals for a period of 5 min by using a SAFAS Flx-Xenius spectrofluorometer. All measurements were performed in triplicate, in the same setting at 25°C. The CS activity was then normalized to the total protein content and was reported in enzyme unit per mg of proteins (U/mg proteins). Proteins were assayed by BCA method.

*2.5.6. Evaluation of phosphatase activities*

For the phosphatase assay, the digestive glands of 10 specimens were  digested and pooled, ground with an Ultra-Turrax apparatus (T25 basic IKA®WERKE), and homogenized with a Thomas® pestle tissue grinder (2min at 200rpm with a JK RW 20n IKA LABORTECHNIK) in a ratio tissue/buffer of 0.2g/1ml containing 100mM Tris-HCl buffer (pH 6.8), 10mM EDTA and 10mM PMSF. Homogenates were centrifuged twice (12200g, 15min, 4°C) and supernatants were filtrated on a 0.45μm filter and stored at -80°C until analysis. The protein concentration in all supernatants was determined using the Bradford method, with bovine serum albumin as the standard protein (3).

Phosphatase activity was measured by Sensolyte kit « pNPP protein phosphatase Assay kit colorimetric » of ANASPEC. Each incubation mixture (100μL) contained 50μL of phosphatases kit buffer with p-NPP and 40µg of digestive gland proteins diluted in 50μL of kit buffer. The samples (i.e. 50μL of gland digestive supernatants corresponding to 40µg of proteins) were incubated for 30min at 37°C. During incubation time, the hydrolysis products were recorded at a wavelength of 405nm on a spectrophotometer (BMG Labtek). Different concentrations of pNP were used as standard and for the quantification of the appeared product of the reaction.  The specific activity of the enzymes was measured in µmol/ml/min/µg (with one unit is defined as the amount of enzyme that catalyses the appearance of 1 µmol of product per min). The data on the specific activity of the phosphatases enzymes were represented as the mean values ± the standard errors (n=3).

**References**

1. Lacroix C, Le Cuff N, Receveur J, Moraga D, Auffret M, Guyomarch J. Development of an innovative and « green » stir bar sorptive extraction-thermal desorption-gas chromatography-tandem mass spectrometry method for quantification of polycyclic aromatic hydrocarbons in marine biota. J Chromatogr A. 4 juill 2014;1349:1‑10.

2. Shrivastava A, Gupta V. Methods for the determination of limit of detection and limit of quantitation of the analytical methods. Chron Young Sci. 1 janv 2011;2(1):21‑5.

3. Bradford MM. A rapid and sensitive method for the quantitation of microgram quantities of protein utilizing the principle of protein-dye binding. Anal Biochem. 7 mai 1976;72:248‑54.

4. Gérard-Monnier D, Erdelmeier I, Régnard K, Moze-Henry N, Yadan JC, Chaudière J. Reactions of 1-methyl-2-phenylindole with malondialdehyde and 4-hydroxyalkenals. Analytical applications to a colorimetric assay of lipid peroxidation. Chem Res Toxicol. oct 1998;11(10):1176‑83.

5. Luna-Acosta A, Rosenfeld E, Amari M, Fruitier-Arnaudin I, Bustamante P, Thomas-Guyon H. First evidence of laccase activity in the Pacific oyster Crassostrea gigas. Fish Shellfish Immunol. avr 2010;28(4):719‑26.

6. Srere PA. [1] Citrate synthase: [EC 4.1.3.7. Citrate oxaloacetate-lyase (CoA-acetylating)]. Lowenstein JM, éditeur. Methods in Enzymology. In Academic Press. New-York: Academic Press; 1969. p. 3‑11.
