# Supplementary material for: Short-Term and Long-Term Biological Effects of Chronic Chemical Contamination on Natural Populations of a Marine Bivalve
Source: PLoS One. 2016 Mar 3;11(3):e0150184. doi: 10.1371/journal.pone.0150184 (PMC4777565; doi:10.1371/journal.pone.0150184)
Supplement: S1 Table — (DOCX) [file pone.0150184.s002.docx]

**Breitwieser, Viricel *et al.* - Supporting Information**

**S1 Table.** Supplementary results for organic contaminant concentrations (µg/Kg of dry weight) in the digestive glands of *Mimahlamys varia* (5<n<10 per sites) from the four sampling areas (Loix-en-Ré, Minimes, Port-Neuf, Les Palles) in March 2014 (Table S1a) and in September 2014 (S1b). LOQ represents Limit of Quantification of organic contaminants in digestive glands of scallops. ND signifies a contamination not be established and LOD represents the limit of detection of organic contaminants (LOQ/3).

| **S2a** | **Contaminants** | **LOQ** (µg/kg of dry weight) | **Loix** | | | | | **Les Minimes** | | | | | **Port-Neuf** | | | | | | | | | | **Les Palles** | | | | |
| --- | --- | --- | --- | --- | --- | --- | --- | --- | --- | --- | --- | --- | --- | --- | --- | --- | --- | --- | --- | --- | --- | --- | --- | --- | --- | --- | --- |
|  |  |  | DG1 | DG2 | DG3 | DG4 | DG5 | DG1 | DG2 | DG3 | DG4 | DG5 | DG1 | DG2 | DG3 | DG4 | DG5 | DG6 | DG7 | DG8 | DG9 | DG10 | DG1 | DG2 | DG3 | DG4 | DG5 |
| **PAH** | benzothiophene | 0.6 | 4.0 | 4.1 | 3.0 | 6.9 | 4.5 | 3.5 | 6.0 | 4.5 | 3.6 | 3.2 | 4.9 | 0.8 | 2.0 | 3.1 | < LOD | < LOD | < LOD | 0.7 | <LOQ | < LOD | 5.6 | 9.9 | 9.1 | 8.0 | 6.9 |
|  | 2-methylnaphtalene | 2.1 | 22.2 | 16.0 | 17.5 | 34.0 | 30.9 | 20.9 | 39.1 | 33.3 | 21.4 | 21.8 | 13.9 | < LOD | <LOQ | 2.8 | < LOD | ND | ND | ND | ND | ND | 32.7 | 62.3 | 54.2 | 53.3 | 38.1 |
|  | biphenyl | 0.5 | 0.9 | 1.6 | 2.0 | 2.6 | 3.4 | 2.2 | 1.5 | 4.1 | 5.1 | 3.6 | 6.1 | 1.4 | 2.0 | 3.0 | 1.8 | < LOD | < LOD | < LOD | 1.2 | < LOD | < LOD | < LOD | < LOD | 1.4 | 17.1 |
|  | Dibenzothiophene | 0.5 | 0.9 | < LOD | < LOD | <LOQ | < LOD | 1.2 | < LOD | < LOD | 3.2 | 3.5 | 42.7 | 7.2 | 39.4 | 44.7 | 18.9 | 1.8 | 2.3 | 3.7 | 3.2 | 1.6 | < LOD | < LOD | < LOD | < LOD | 28.4 |
|  | Phenanthrene | 1.5 | 74.9 | 26.5 | 31.4 | 52.2 | 47.6 | 67.9 | 71.5 | 20.6 | 103.6 | 86.9 | 3.7 | 1.7 | 3.7 | 7.4 | 2.8 | 18.9 | 23.6 | 31.5 | 46.2 | 12.6 | < LOD | 5.6 | < LOD | < LOD | 493.9 |
|  | 2methylfluoranthene | 1.1 | 11.2 | 3.9 | 4.3 | 9.2 | 7.0 | 16.3 | 19.7 | 8.5 | 85.0 | 15.0 | 55.6 | 9.6 | 23.1 | 84.9 | 42.4 | ND | ND | ND | ND | ND | 6.5 | 13.2 | 3.0 | 2.5 | 103.4 |
|  | benzoaanthracene | 1.3 | 53.4 | 19.3 | 16.5 | 45.5 | 35.1 | 118.1 | 115.2 | 54.4 | 588.1 | 106.1 | 7.3 | < LOD | < LOD | 67.7 | 17.8 | 9.3 | 9.1 | 19.9 | 56.7 | 13.6 | 33.2 | 72.4 | 18.2 | 28.7 | 411.9 |
|  | chrysene | 0.7 | 80.7 | 32.5 | 39.9 | 72.6 | 65.0 | 173.3 | 139.0 | 68.6 | 782.4 | 141.5 | 30.4 | 4.6 | 25.7 | 41.2 | 23.1 | 42.4 | 49.6 | 49.6 | 84.9 | 23.6 | 42.0 | 82.1 | 14.0 | 33.1 | 427.5 |
|  | benzobfluoranthene | 0.6 | 47.8 | 25.5 | 14.5 | 56.7 | 34.1 | 97.9 | 95.2 | 51.5 | 598.6 | 76.2 | < LOD | < LOD | < LOD | 38.5 | 11.4 | 17.8 | 50.8 | 80.0 | 89.6 | 34.9 | 29.7 | 66.2 | 24.1 | 14.7 | 311.9 |
|  | benzokfluoranthene | 0.7 | 69.1 | 31.9 | 27.9 | 71.1 | 52.0 | 156.4 | 161.3 | 76.7 | 586.6 | 137.9 | 18.7 | 2.8 | 14.1 | 30.8 | 2.9 | 23.1 | 48.7 | 20.1 | 33.1 | 20.6 | 58.2 | 93.2 | 47.8 | 45.9 | 409.5 |
|  | benzoepyrene | 0.5 | 43.5 | 18.1 | 16.4 | 46.1 | 32.2 | 93.5 | 98.7 | 62.2 | 393.2 | 95.8 | 10.2 | < LOD | < LOD | 28.0 | 7.2 | 11.4 | 34.5 | 25.3 | 35.2 | 15.1 | 38.7 | 77.8 | 30.7 | 34.3 | 293.9 |
|  | benzoapyrene | 0.7 | 57.5 | 22.1 | 15.3 | 55.8 | 40.1 | 98.5 | 110.3 | 58.9 | 413.6 | 92.7 | < LOD | < LOD | < LOD | < LOD | < LOD | 2.9 | 1.7 | 3.3 | 28.9 | 13.1 | 40.8 | 69.0 | 27.9 | 28.4 | 359.6 |
|  | perylene | 0.5 | 25.7 | 15.0 | 14.7 | 30.7 | 21.9 | 72.0 | 52.7 | 35.0 | 169.7 | 56.9 | < LOD | < LOD | < LOD | < LOD | < LOD | 7.2 | 29.6 | 21.4 | 43.6 | 58.8 | 36.2 | 53.8 | 44.0 | 35.0 | 137.0 |
|  | indeno123cdpyrene | 6.3 | 66.4 | 18.7 | 13.8 | 49.9 | 44.3 | 57.5 | 88.2 | 37.4 | 127.2 | 86.5 | < LOD | < LOD | < LOD | < LOD | < LOD | < LOD | < LOD | < LOD | < LOD | < LOD | 8.1 | 43.4 | < LOD | 7.8 | 275.9 |
|  | dibenzoahanthracene | 10.0 | < LOD | < LOD | < LOD | < LOD | < LOD | 35.2 | 33.8 | < LOD | 100.0 | 70.6 | ND | ND | ND | ND | ND | < LOD | < LOD | < LOD | < LOD | < LOD | < LOD | < LOD | < LOD | < LOD | 219.9 |
|  | bpe | 2.9 | 55.1 | 8.4 | 6.8 | 40.5 | 40.6 | 56.8 | 88.8 | 28.8 | 113.4 | 91.6 | ND | ND | ND | ND | ND | < LOD | < LOD | < LOD | < LOD | < LOD | 16.2 | 28.2 | 17.4 | < LOD | 244.9 |
| **PCB** | PCB 7 | 1.0 | <LOQ | <LOQ | < LOD | <LOQ | <LOQ | <LOQ | <LOQ | <LOQ | <LOQ | <LOQ | ND | ND | ND | ND | ND | ND | ND | ND | ND | ND | <LOQ | 1.0 | 1.0 | 1.0 | <LOQ |
|  | PCB 28 | 1.0 | <LOQ | <LOQ | <LOQ | <LOQ | <LOQ | 2.0 | 1.3 | <LOQ | 3.2 | 1.2 | ND | ND | ND | ND | ND | ND | ND | ND | ND | ND | <LOQ | <LOQ | <LOQ | <LOQ | <LOQ |
|  | PCB 52 | 1.0 | <LOQ | < LOD | <LOQ | < LOD | <LOQ | 9.5 | <LOQ | <LOQ | 14.6 | <LOQ | ND | ND | ND | ND | ND | ND | ND | ND | ND | ND | <LOQ | <LOQ | <LOQ | <LOQ | <LOQ |
|  | PCB 35 | 1.0 | <LOQ | <LOQ | <LOQ | <LOQ | <LOQ | <LOQ | <LOQ | <LOQ | 1.2 | <LOQ | ND | ND | ND | ND | ND | ND | ND | ND | ND | ND | <LOQ | 1.5 | 1.4 | 1.1 | 1.4 |
|  | PCB 101 | 1.0 | < LOD | 1.1 | 1.8 | < LOD | < LOD | 55.3 | < LOD | < LOD | 73.7 | 3.3 | ND | ND | ND | ND | ND | ND | ND | ND | ND | ND | < LOD | < LOD | < LOD | < LOD | < LOD |
|  | PCB 77 | 1.0 | <LOQ | <LOQ | <LOQ | <LOQ | <LOQ | 1.5 | <LOQ | <LOQ | 3.0 | <LOQ | ND | ND | ND | ND | ND | ND | ND | ND | ND | ND | <LOQ | <LOQ | <LOQ | <LOQ | <LOQ |
|  | PCB 135 | 1.0 | < LOD | < LOD | <LOQ | < LOD | <LOQ | 8.2 | 1.5 | < LOD | 10.7 | 2.4 | ND | ND | ND | ND | ND | ND | ND | ND | ND | ND | < LOD | < LOD | < LOD | < LOD | < LOD |
|  | PCB 118 | 1.0 | 1.4 | 1.0 | 4.0 | 1.1 | 3.3 | 38.3 | 11.2 | 1.1 | 37.2 | 11.8 | ND | ND | ND | ND | ND | ND | ND | ND | ND | ND | < LOD | < LOD | < LOD | < LOD | < LOD |
|  | PCB 105 | 1.0 | <LOQ | <LOQ | <LOQ | <LOQ | <LOQ | 5.8 | 1.4 | < LOD | 6.4 | 2.0 | ND | ND | ND | ND | ND | ND | ND | ND | ND | ND | < LOD | < LOD | < LOD | < LOD | < LOD |
|  | PCB 138 | 1.0 | 6.8 | 6.6 | 15.3 | 6.2 | 12.9 | 91.5 | 33.3 | 3.2 | 74.2 | 33.6 | ND | ND | ND | ND | ND | ND | ND | ND | ND | ND | < LOD | < LOD | < LOD | 8.9 | < LOD |
|  | PCB 156 | 1.0 | < LOD | < LOD | < LOD | < LOD | < LOD | <LOQ | < LOD | < LOD | <LOQ | <LOQ | ND | ND | ND | ND | ND | ND | ND | ND | ND | ND | < LOD | < LOD | < LOD | < LOD | < LOD |
|  | PCB 169 | 1.0 | < LOD | < LOD | < LOD | < LOD | < LOD | < LOD | < LOD | < LOD | <LOQ | <LOQ | ND | ND | ND | ND | ND | ND | ND | ND | ND | ND | < LOD | < LOD | < LOD | < LOD | < LOD |
|  | hexachlorobenzene | 1.0 | < LOD | < LOD | < LOD | < LOD | < LOD | < LOD | < LOD | 1.7 | <LOQ | < LOD | ND | ND | ND | ND | ND | ND | ND | ND | ND | ND | < LOD | < LOD | < LOD | < LOD | < LOD |
|  | metolachlore | 2.5 | < LOD | < LOD | < LOD | < LOD | < LOD | < LOD | < LOD | < LOD | < LOD | < LOD | ND | ND | ND | ND | ND | ND | ND | ND | ND | ND | < LOD | < LOD | < LOD | < LOD | < LOD |
| **Pesticides** | isodrine | 1.0 | < LOD | < LOD | < LOD | < LOD | < LOD | < LOD | < LOD | < LOD | < LOD | < LOD | ND | ND | ND | ND | ND | ND | ND | ND | ND | ND | < LOD | < LOD | < LOD | < LOD | < LOD |
|  | 2-4-dde | 1.0 | < LOD | < LOD | < LOD | < LOD | < LOD | < LOD | < LOD | < LOD | < LOD | < LOD | ND | ND | ND | ND | ND | ND | ND | ND | ND | ND | < LOD | < LOD | < LOD | < LOD | < LOD |
|  | 4-4-dde | 1.0 | 1.7 | 1.2 | 2.7 | 1.7 | 2.8 | 11.8 | 5.8 | <LOQ | 18.2 | 6.4 | ND | ND | ND | ND | ND | ND | ND | ND | ND | ND | 1.3 | 1.3 | < LOD | 1.7 | 1.4 |
|  | endrine | 2.0 | < LOD | < LOD | < LOD | < LOD | < LOD | < LOD | < LOD | < LOD | < LOD | < LOD | ND | ND | ND | ND | ND | ND | ND | ND | ND | ND | < LOD | < LOD | < LOD | < LOD | < LOD |

| **S2b** | **Contaminants** | **LOQ**  (µg/kg of dry weight) | **Loix** | | | | | | | | | | **Les Minimes** | | | | | | | | | |
| --- | --- | --- | --- | --- | --- | --- | --- | --- | --- | --- | --- | --- | --- | --- | --- | --- | --- | --- | --- | --- | --- | --- |
|  |  |  | DG1 | DG2 | DG3 | DG4 | DG5 | DG6 | DG7 | DG8 | DG9 | DG10 | DG1 | DG2 | DG3 | DG4 | DG5 | DG6 | DG7 | DG8 | DG9 | DG10 |
| **PAH** | benzothiophene | 28 | <LOD | <LOD | <LOD | <LOD | <LOD | <LOD | <LOD | <LOD | <LOQ | <LOD | <LOD | <LOD | <LOD | <LOD | <LOD | <LOD | <LOD | <LOD | <LOD | <LOD |
|  | biphenyl | 28 | 38.81 | <LOQ | 36.33 | <LOQ | 31.31 | 47.98 | 26.21 | <LOQ | 20.29 | 44.63 | <LOQ | <LOD | <LOQ | <LOQ | 68.89 | <LOQ | 38.88 | <LOQ | <LOQ | <LOQ |
|  | Dibenzothiophene | 14 | <LOD | <LOD | <LOD | <LOD | <LOD | <LOD | <LOD | <LOD | <LOD | <LOD | <LOD | <LOD | <LOD | <LOD | <LOD | <LOD | <LOD | <LOD | <LOD | <LOD |
|  | Phenanthrene | 28 | <LOQ | <LOQ | <LOQ | <LOQ | <LOQ | <LOQ | <LOQ | <LOQ | <LOQ | <LOQ | <LOQ | <LOQ | <LOQ | <LOQ | <LOQ | <LOQ | <LOQ | 29.77 | <LOQ | 28.36 |
|  | benzo(a)anthracene | 2.8 | 30.33 | 47.46 | 23.79 | 21.93 | 17.92 | 29.79 | 18.01 | 8.89 | 27.13 | 12.28 | 25.74 | 10.57 | 54.96 | 27.66 | 64.08 | 20.82 | 105.68 | 93.91 | 23.39 | 26.63 |
|  | chrysene | 2.8 | 53.80 | 71.70 | 40.77 | 22.78 | 45.31 | 61.34 | 39.27 | 22.79 | 52.06 | 25.26 | 50.33 | 19.83 | 81.66 | 49.52 | 95.34 | 40.36 | 165.15 | 132.43 | 44.37 | 61.05 |
|  | benzo(b)fluoranthene | ND | ND | ND | ND | ND | ND | ND | ND | ND | ND | ND | ND | ND | ND | ND | ND | ND | ND | ND | ND | ND |
|  | benzo(k)fluoranthene | ND | ND | ND | ND | ND | ND | ND | ND | ND | ND | ND | ND | ND | ND | ND | ND | ND | ND | ND | ND | ND |
|  | benzo(b+k)fluoranthene | 5.6 | 120.36 | 161.53 | 71.86 | 100.30 | 60.99 | 156.90 | 65.82 | 35.73 | 90.50 | 64.64 | 88.87 | 47.14 | 187.02 | 82.81 | 170.94 | 68.90 | 168.86 | 240.66 | 87.18 | 80.15 |
|  | benzoepyrene | 14 | 34.75 | 43.42 | 19.41 | 65.97 | 23.12 | 39.76 | 21.26 | 15.24 | 29.34 | 20.30 | 31.18 | 18.24 | 49.56 | 29.83 | 49.05 | 27.66 | 63.09 | 70.59 | 26.57 | 25.20 |
|  | benzoapyrene | 2.8 | 23.79 | 41.47 | 13.93 | 46.42 | 13.75 | 24.06 | 12.99 | 5.67 | 21.64 | 11.37 | 21.86 | 8.42 | 39.83 | 19.81 | 41.56 | 13.31 | 51.50 | 60.71 | 16.57 | 12.91 |
|  | perylene | 2.8 | 24.17 | 26.58 | 15.01 | 60.68 | 15.12 | 30.19 | 15.68 | 12.29 | 20.65 | 19.63 | 27.16 | 20.22 | 37.75 | 26.53 | 39.51 | 22.79 | 33.48 | 47.57 | 25.19 | 23.07 |
|  | indeno123cdpyrene | 14 | 13.20 | 22.75 | <LOQ | 61.87 | 12.87 | 16.37 | 14.60 | <LOQ | 18.79 | 13.70 | 18.72 | 9.62 | 15.85 | 12.59 | 15.54 | 13.27 | 14.82 | 17.17 | <LOQ | <LOQ |
|  | dibenzoahanthracene | 14 | <LOQ | <LOQ | <LOQ | 58.55 | <LOQ | <LOQ | <LOQ | <LOQ | <LOQ | <LOQ | <LOQ | <LOQ | <LOQ | <LOQ | <LOQ | <LOQ | <LOQ | <LOQ | <LOQ | <LOQ |
|  | benzo(ghi)perylene | 2.8 | 9.13 | 15.72 | 7.75 | 65.78 | 10.02 | 12.18 | 10.55 | 7.92 | 14.95 | 9.14 | 15.60 | 7.93 | 12.89 | 11.35 | 13.60 | 10.87 | 13.12 | 14.44 | 10.00 | 7.12 |
| **PCB** | PCB 7 | 2.8 | <LOD | <LOD | <LOD | <LOD | <LOD | <LOD | <LOD | <LOD | <LOD | <LOD | <LOD | <LOD | <LOD | <LOD | <LOD | <LOD | <LOD | <LOD | <LOD | <LOD |
|  | PCB 28 | 2.8 | <LOD | <LOD | <LOD | <LOD | <LOD | <LOD | <LOD | <LOD | <LOD | <LOD | <LOQ | <LOQ | <LOQ | <LOQ | <LOQ | <LOQ | <LOQ | <LOQ | <LOQ | <LOQ |
|  | PCB 52 | 2.8 | <LOD | <LOD | <LOD | <LOD | <LOD | <LOD | <LOD | <LOD | <LOQ | <LOD | <LOQ | <LOQ | 6.78 | 4.67 | 5.76 | <LOQ | 12.54 | 5.26 | 8.46 | 7.98 |
|  | PCB 35 | 2.8 | <LOD | <LOD | <LOD | <LOD | <LOD | <LOD | <LOD | <LOD | <LOD | <LOD | <LOD | <LOD | <LOD | <LOD | <LOD | <LOD | <LOD | <LOD | <LOD | <LOD |
|  | PCB 101 | 14 | <LOD | <LOD | <LOD | <LOD | <LOQ | <LOQ | <LOD | <LOD | <LOQ | <LOD | <LOQ | <LOQ | 20.53 | 14.15 | 19.89 | <LOQ | 26.99 | 24.85 | 15.64 | 33.28 |
|  | PCB 77 | 2.8 | <LOD | <LOD | <LOD | <LOD | <LOD | <LOD | <LOD | <LOD | <LOD | <LOD | <LOD | <LOD | <LOD | <LOD | <LOD | <LOD | <LOD | <LOQ | <LOD | <LOQ |
|  | PCB 135 | 2.8 | <LOQ | <LOQ | <LOQ | <LOD | <LOQ | <LOQ | <LOQ | <LOQ | <LOQ | <LOD | 3.17 | <LOQ | 4.73 | 3.09 | 4.61 | <LOQ | 5.25 | 7.47 | 2.94 | 7.98 |
|  | PCB 118 | 2.8 | 7.37 | 7.40 | 6.82 | <LOQ | 8.69 | 12.98 | 8.87 | 7.18 | 8.76 | 4.37 | 15.37 | 8.66 | 21.71 | 14.71 | 21.81 | 14.31 | 25.21 | 33.64 | 13.96 | 34.42 |
|  | PCB 105 | 14 | <LOD | <LOD | <LOD | <LOD | <LOD | <LOD | <LOD | <LOD | <LOD | <LOD | <LOQ | <LOD | <LOQ | <LOQ | <LOQ | <LOD | <LOQ | <LOQ |  | <LOQ |
|  | PCB 138 | 14 | 20.90 | 21.77 | 20.45 | <LOQ | 25.55 | 38.92 | 27.07 | 21.85 | 25.91 | 15.03 | 37.05 | 22.16 | 47.82 | 32.34 | 46.91 | 30.55 | 53.60 | 75.85 | 30.01 | 77.43 |
|  | PCB 156 | 14 | <LOD | <LOD | <LOD | <LOD | <LOD | <LOD | <LOD | <LOD | <LOD | <LOD | <LOD | <LOD | <LOD | <LOD | <LOD | <LOD | <LOD | <LOD | <LOD | <LOD |
|  | PCB 169 | 14 | <LOD | <LOD | <LOD | <LOD | <LOD | <LOD | <LOD | <LOD | <LOD | <LOD | <LOD | <LOD | <LOD | <LOD | <LOD | <LOD | <LOD | <LOD | <LOD | <LOD |
|  | hexachlorobenzene | 14 | <LOD | <LOD | <LOD | <LOD | <LOD | <LOD | <LOD | <LOD | <LOD | <LOD | <LOD | <LOD | <LOD | <LOD | <LOD | <LOD | <LOD | <LOD | <LOD | <LOD |
|  | metolachlore | 2.8 | <LOD | <LOD | <LOD | <LOD | <LOD | <LOD | <LOD | <LOD | <LOD | <LOD | <LOD | <LOD | <LOD | <LOD | <LOD | <LOD | <LOD | <LOD | <LOD | <LOD |
| **Pesticides** | isodrine | 14 | <LOD | <LOD | <LOD | <LOD | <LOD | <LOD | <LOD | <LOD | <LOD | <LOD | <LOD | <LOD | <LOD | <LOD | <LOD | <LOD | <LOD | <LOD | <LOD | <LOD |
|  | 2-4-dde | 14 | <LOD | <LOD | <LOD | <LOD | <LOD | <LOD | <LOD | <LOD | <LOD | <LOD | <LOD | <LOD | <LOD | <LOD | <LOD | <LOD | <LOD | <LOD | <LOD | <LOD |
|  | 4-4-dde | 14 | 3.13 | 3.35 | 2.82 | <LOD | 4.59 | 5.01 | 4.30 | 2.88 | 4.13 | <LOQ | 4.89 | 2.09 | 6.48 | 4.24 | 5.39 | 3.22 | 7.39 | 10.70 | 4.27 | 10.86 |
|  | endrine | 2.8 | <LOD | <LOD | <LOD | <LOD | <LOD | <LOD | <LOD | <LOD | <LOD | <LOD | <LOD | <LOD | <LOD | <LOD | <LOD | <LOD | <LOD | <LOD | <LOD | <LOD |
|  |  |  |  |  |  |  |  |  |  |  |  |  |  |  |  |  |  |  |  |  |  |  |
| **S2b con’t** | **Contaminants** | **LOQ**  (µg/kg of dry weight) | **Port-Neuf** | | | | | | | | | | **Les Palles** | | | | | | | | | |
|  |  |  | DG1 | DG2 | DG3 | DG4 | DG5 | DG6 | DG7 | DG8 | DG9 | DG10 | DG1 | DG2 | DG3 | DG4 | DG5 | DG6 | DG7 | DG8 | DG9 | DG10 |
| **PAH** | benzothiophene | 28 | rep | <LOD | <LOD | <LOD | <LOD | <LOD | <LOD | <LOD | <LOD | <LOD | <LOD | <LOD | <LOD | <LOD | <LOD | <LOD | <LOD | <LOD | <LOD | <LOD |
|  | biphenyl | 28 | 24.30 | 28.71 | <LOQ | 30.68 | 17.25 | 27.24 | <LOQ | 54.94 | 24.01 | 26.16 | <LOQ | <LOQ | 39.96 | <LOQ | <LOQ | <LOQ | 31.31 | <LOQ | 41.36 | <LOQ |
|  | Dibenzothiophene | 14 | <LOD | <LOD | <LOD | <LOD | <LOD | <LOD | <LOD | <LOD | <LOD | <LOD | <LOD | <LOD | <LOD | <LOD | <LOQ | <LOD | <LOD | <LOQ | <LOD | <LOQ |
|  | Phenanthrene | 28 | <LOQ | 27.92 | 23.51 | <LOQ | <LOQ | 24.80 | <LOQ | 34.11 | <LOQ | <LOQ | 22.94 | 32.89 | <LOQ | 44.52 | 111.71 | <LOQ | 24.71 | 22.66 | 32.09 | 93.82 |
|  | benzo(a)anthracene | 2.8 | 17.44 | 43.43 | 22.80 | 16.88 | 16.71 | 27.25 | 27.01 | 27.41 | 33.89 | 35.98 | 60.55 | 38.48 | 44.85 | 102.59 | 179.42 | 38.05 | 109.01 | 34.92 | 103.46 | 126.97 |
|  | chrysene | 2.8 | 32.97 | 66.57 | 49.43 | 35.70 | 31.24 | 57.68 | 48.68 | 56.12 | 60.12 | 55.70 | 77.12 | 61.56 | 66.79 | 133.31 | 225.87 | 60.56 | 129.80 | 65.53 | 127.61 | 171.19 |
|  | benzo(b)fluoranthene | ND | ND | ND | ND | ND | ND | ND | ND | ND | ND | ND | ND | ND | ND | ND | ND | ND | ND | ND | ND | ND |
|  | benzo(k)fluoranthene | ND | ND | ND | ND | ND | ND | ND | ND | ND | ND | ND | ND | ND | ND | ND | ND | ND | ND | ND | ND | ND |
|  | benzo(b+k)fluoranthene | 5.6 | 80.58 | 96.75 | 47.39 | 58.58 | 45.10 | 79.34 | 90.65 | 84.12 | 96.15 | 112.85 | 127.97 | 99.71 | 102.04 | 253.41 | 377.85 | 83.00 | 225.80 | 92.47 | 157.91 | 302.50 |
|  | benzoepyrene | 14 | 26.96 | 30.26 | 17.18 | 19.49 | 16.32 | 27.26 | 28.56 | 28.64 | 26.81 | 32.92 | 41.48 | 39.50 | 37.89 | 76.00 | 129.65 | 27.30 | 64.92 | 34.92 | 48.91 | 98.51 |
|  | benzoapyrene | 2.8 | 13.71 | 24.14 | 9.29 | 13.87 | 11.19 | 19.10 | 21.97 | 22.53 | 22.21 | 23.00 | 42.94 | 31.98 | 26.08 | 79.95 | 153.69 | 24.21 | 82.66 | 20.55 | 55.04 | 125.41 |
|  | perylene | 2.8 | 28.66 | 22.70 | 12.21 | 17.78 | 13.44 | 17.30 | 23.16 | 20.73 | 24.70 | 24.66 | 38.92 | 37.34 | 39.01 | 58.75 | 86.66 | 31.51 | 50.86 | 38.15 | 46.47 | 75.50 |
|  | indeno123cdpyrene | 14 | 13.02 | <LOQ | <LOQ | <LOQ | <LOQ | <LOQ | <LOQ | <LOQ | <LOQ | <LOQ | 26.26 | 21.58 | 13.58 | 28.67 | 83.10 | 12.21 | 30.33 | 12.29 | 18.43 | 90.94 |
|  | dibenzoahanthracene | 14 | <LOQ | <LOQ | <LOD | <LOQ | <LOQ | <LOQ | <LOQ | <LOQ | <LOQ | <LOQ | 12.10 | 11.84 | <LOQ | 11.15 | 33.28 | <LOQ | 15.81 | <LOQ | <LOQ | 38.75 |
|  | benzo(ghi)perylene | 2.8 | 12.28 | 9.87 | 4.73 | 11.34 | 8.07 | 10.94 | 11.13 | 17.74 | 11.97 | 8.97 | 19.44 | 18.36 | 11.79 | 21.32 | 61.48 | 10.77 | 22.81 | 11.83 | 13.71 | 63.42 |
| **PCB** | PCB 7 | 2.8 | <LOD | <LOD | <LOD | <LOD | <LOD | <LOD | <LOD | <LOD | <LOD | <LOD | <LOD | <LOD | <LOD | <LOD | <LOD | <LOD | <LOD | <LOD | <LOD | <LOD |
|  | PCB 28 | 2.8 | <LOQ | <LOQ | <LOQ | <LOQ | <LOQ | <LOQ | <LOQ | <LOQ | <LOQ | <LOQ | <LOD | <LOD | <LOD | <LOQ | <LOQ | <LOD | <LOD | <LOQ | <LOD | <LOQ |
|  | PCB 52 | 2.8 | 5.48 | 19.21 | 19.75 | 8.36 | 3.43 | 9.87 | 4.20 | 10.99 | 4.11 | <LOQ | ND | <LOQ | <LOD | <LOD | <LOD | <LOQ | <LOD | <LOD | <LOQ | <LOQ |
|  | PCB 35 | 2.8 | <LOD | <LOD | <LOD | <LOD | <LOD | <LOD | <LOD | <LOD | <LOD | <LOD | <LOD | <LOD | <LOD | <LOD | <LOD | <LOD | <LOD | <LOD | <LOD | <LOD |
|  | PCB 101 | 14 | 17.88 | 34.93 | 43.25 | 17.74 | 13.96 | 38.87 | 18.67 | 34.84 | 19.92 | 14.82 | <LOQ | <LOQ | <LOQ | 12.33 | <LOQ | <LOQ | <LOQ | <LOQ | <LOQ | <LOQ |
|  | PCB 77 | 2.8 | <LOD | <LOQ | <LOQ | <LOD | <LOD | <LOQ | <LOQ | <LOQ | <LOD | <LOD | <LOD | <LOD | <LOD | <LOD | <LOD | <LOD | <LOD | <LOD | <LOD | <LOD |
|  | PCB 135 | 2.8 | 3.13 | 4.78 | 6.49 | 2.55 | 2.41 | 7.39 | 3.92 | 5.90 | 4.10 | 4.06 | <LOQ | 3.21 | <LOQ | 3.92 | 3.65 | 2.61 | <LOQ | 4.44 | 3.22 | 3.06 |
|  | PCB 118 | 2.8 | 14.99 | 31.37 | 39.88 | 18.99 | 17.98 | 43.23 | 24.94 | 36.99 | 27.44 | 27.56 | 5.83 | 9.30 | 5.68 | 12.37 | 11.44 | 7.69 | 5.03 | 12.59 | 10.43 | 9.22 |
|  | PCB 105 | 14 | <LOQ | <LOQ | <LOQ | <LOQ | <LOQ | <LOQ | <LOQ | <LOQ | <LOQ | <LOQ | <LOD | <LOD | <LOD | <LOQ | <LOQ | <LOD | <LOD | <LOQ | <LOD | <LOD |
|  | PCB 138 | 14 | 34.56 | 53.42 | 71.97 | 30.22 | 28.24 | 86.74 | 41.14 | 60.33 | 45.75 | 51.25 | 21.11 | 37.99 | 23.39 | 42.66 | 38.42 | 28.03 | 18.22 | 46.09 | 35.88 | 32.40 |
|  | PCB 156 | 14 | <LOD | <LOD | <LOD | <LOD | <LOD | <LOD | <LOD | <LOD | <LOD | <LOD | <LOD | <LOD | <LOD | <LOD | <LOD | <LOD | <LOD | <LOD | <LOD | <LOD |
|  | PCB 169 | 14 | <LOD | <LOD | <LOD | <LOD | <LOD | <LOD | <LOD | <LOD | <LOD | <LOD | <LOD | <LOD | <LOD | <LOD | <LOD | <LOD | <LOD | <LOD | <LOD | <LOD |
|  | hexachlorobenzene | 14 | <LOD | <LOD | <LOD | <LOD | <LOD | <LOD | <LOD | <LOD | <LOD | <LOD | <LOD | <LOD | <LOD | <LOD | <LOD | <LOD | <LOD | <LOD | <LOD | <LOD |
|  | metolachlore | 2.8 | <LOD | <LOD | <LOD | <LOD | <LOD | <LOD | <LOD | <LOD | <LOD | <LOD | <LOD | <LOD | <LOD | <LOD | <LOD | <LOD | <LOD | <LOD | <LOD | <LOD |
| **Pesticides** | isodrine | 14 | <LOD | <LOD | <LOD | <LOD | <LOD | <LOD | <LOD | <LOD | <LOD | <LOD | <LOD | <LOD | <LOD | <LOD | <LOD | <LOD | <LOD | <LOD | <LOD | <LOD |
|  | 2-4-dde | 14 | <LOD | <LOD | <LOD | <LOD | <LOD | <LOD | <LOD | <LOD | <LOD | <LOD | <LOD | <LOD | <LOD | <LOD | <LOD | <LOD | <LOD | <LOD | <LOD | <LOD |
|  | 4-4-dde | 14 | 3.62 | 4.77 | 7.64 | <LOQ | <LOQ | 7.32 | 3.83 | 4.63 | 3.20 | 4.06 | 2.33 | 4.83 | 2.87 | 5.67 | 4.34 | 4.61 | <LOQ | 6.56 | 4.86 | 4.66 |
|  | endrine | 2.8 | <LOD | <LOD | <LOD | <LOD | <LOD | <LOD | <LOD | <LOD | <LOD | <LOD | <LOD | <LOD | <LOD | <LOD | <LOD | <LOD | <LOD | <LOD | <LOD | <LOD |
|  |  |  |  |  |  |  |  |  |  |  |  |  |  |  |  |  |  |  |  |  |  |  |
