# Supplementary material for: Short-Term and Long-Term Biological Effects of Chronic Chemical Contamination on Natural Populations of a Marine Bivalve
Source: PLoS One. 2016 Mar 3;11(3):e0150184. doi: 10.1371/journal.pone.0150184 (PMC4777565; doi:10.1371/journal.pone.0150184)
Supplement: S2 Table — (DOCX) [file pone.0150184.s003.docx]

**Breitwieser, Viricel *et al.* - Supporting Information**

**S2 Table. Individual data for biomarker levels (S2a) and metal concentrations (S2b).**

**S2a**

| **date** | **sampling site** | **individual** | **CS** | **MDA** | **SOD** | **GST** | **LACCASE** |
| --- | --- | --- | --- | --- | --- | --- | --- |
| march | Loix | 1 | 0,04 | 175,41 | 33,14 | 2733,54 | 4,17 |
| march | Loix | 2 | 0,03 | 123,93 | 35,25 | 2157,29 | 4,00 |
| march | Loix | 3 | 0,02 | 123,72 | 34,78 | 3106,32 | 2,25 |
| march | Loix | 4 | 0,02 | 183,73 | 33,06 | 3000,58 | 3,35 |
| march | Loix | 5 | 0,03 | 79,12 | 36,02 | 2850,93 | 4,48 |
| march | Loix | 6 | 0,02 | 91,66 | 29,27 | 2859,92 | 4,91 |
| march | Loix | 7 | 0,03 | 110,16 | 36,36 | 2708,42 | 4,57 |
| march | Loix | 8 | 0,03 | 87,96 | 37,32 | 2270,97 | 3,50 |
| march | Loix | 9 | NA | 143,24 | 36,99 | 3263,77 | 4,06 |
| march | Loix | 10 | 0,02 | 94,74 | 34,52 | 3617,75 | 3,81 |
| march | Port-Neuf | 1 | 0,01 | 79,84 | 50,24 | 2751,90 | 4,60 |
| march | Port-Neuf | 2 | 0,01 | 132,25 | 53,54 | 2538,43 | 3,65 |
| march | Port-Neuf | 3 | 0,02 | 112,52 | 51,02 | 3247,88 | 3,26 |
| march | Port-Neuf | 4 | 0,02 | 151,05 | 52,82 | 3174,16 | 4,30 |
| march | Port-Neuf | 5 | 0,02 | 245,80 | 46,75 | 3470,03 | 4,06 |
| march | Port-Neuf | 6 | 0,02 | 134,61 | 52,16 | 3695,39 | 2,96 |
| march | Port-Neuf | 7 | 0,02 | 167,29 | 54,39 | 4088,85 | 4,08 |
| march | Port-Neuf | 8 | 0,02 | 116,53 | 56,73 | 4017,79 | 4,23 |
| march | Port-Neuf | 9 | 0,02 | 114,88 | 46,21 | 2973,23 | 4,50 |
| march | Port-Neuf | 10 | 0,02 | 112,21 | 57,51 | 3599,74 | 3,23 |
| march | Minimes | 1 | 0,03 | 323,27 | 35,71 | 2100,79 | 10,09 |
| march | Minimes | 2 | 0,02 | 130,91 | 39,86 | 2505,79 | 8,66 |
| march | Minimes | 3 | 0,02 | 154,14 | 37,43 | 2765,77 | 5,71 |
| march | Minimes | 4 | 0,02 | 90,84 | 40,33 | 1951,37 | 9,76 |
| march | Minimes | 5 | 0,02 | 143,45 | 45,29 | 2952,93 | 5,11 |
| march | Minimes | 6 | 0,02 | 125,77 | 35,74 | 2981,58 | 9,49 |
| march | Minimes | 7 | 0,02 | 231,61 | 45,28 | 2950,35 | 13,95 |
| march | Minimes | 8 | 0,01 | 108,10 | 35,19 | 2033,10 | 4,80 |
| march | Minimes | 9 | 0,03 | 105,63 | 38,50 | 2768,64 | 20,55 |
| march | Minimes | 10 | 0,03 | 102,96 | 35,83 | 2536,20 | 17,51 |
| march | Les Palles | 1 | 0,01 | 112,42 | 44,55 | 2604,63 | 5,54 |
| march | Les Palles | 2 | 0,02 | 124,95 | 35,29 | 1573,11 | 4,66 |
| march | Les Palles | 3 | 0,02 | 112,01 | 45,25 | 2128,54 | 6,22 |
| march | Les Palles | 4 | 0,01 | 134,61 | 40,98 | 2159,38 | 4,19 |
| march | Les Palles | 5 | 0,03 | 100,29 | NA | 1906,40 | 5,94 |
| march | Les Palles | 6 | 0,02 | 159,27 | 42,02 | 2152,51 | 4,77 |
| march | Les Palles | 7 | 0,03 | 153,31 | 31,69 | 2543,71 | 3,98 |
| march | Les Palles | 8 | 0,02 | 89,40 | 34,72 | 2369,35 | 4,14 |
| march | Les Palles | 9 | 0,02 | 125,36 | 40,95 | 2642,34 | 5,00 |
| march | Les Palles | 10 | 0,02 | 142,63 | 33,22 | 2411,42 | 3,72 |
| sept | Loix | 1 | 0,03 | 100,76 | NA | 1796,88 | 3,47 |
| sept | Loix | 2 | 0,03 | 93,97 | 53,85 | 1882,81 | NA |
| sept | Loix | 3 | 0,02 | 95,17 | 62,85 | 1614,19 | 2,98 |
| sept | Loix | 4 | 0,01 | 68,12 | 48,21 | 1605,03 | 2,99 |
| sept | Loix | 5 | 0,02 | 37,02 | 53,48 | 1196,19 | 4,28 |
| sept | Loix | 6 | 0,02 | 72,12 | 61,74 | 1498,90 | 2,93 |
| sept | Loix | 7 | 0,02 | 108,75 | 57,59 | NA | 3,97 |
| sept | Loix | 8 | 0,02 | 139,14 | 60,60 | 1573,73 | 4,50 |
| sept | Loix | 9 | 0,01 | 86,08 | 60,37 | 1973,45 | 2,39 |
| sept | Loix | 10 | 0,02 | 127,92 | 56,86 | 3100,95 | 2,99 |
| sept | Port-Neuf | 1 | 0,04 | 212,31 | 54,31 | 1825,13 | 3,95 |
| sept | Port-Neuf | 2 | 0,02 | 115,91 | 57,82 | 2538,44 | NA |
| sept | Port-Neuf | 3 | 0,02 | 176,65 | 67,97 | 1040,19 | 3,78 |
| sept | Port-Neuf | 4 | 0,01 | 106,22 | 54,90 | 2257,47 | 3,35 |
| sept | Port-Neuf | 5 | 0,01 | 137,27 | 52,76 | 2342,65 | 4,27 |
| sept | Port-Neuf | 6 | 0,01 | 160,01 | NA | 1940,61 | 3,94 |
| sept | Port-Neuf | 7 | 0,01 | 151,19 | 57,59 | 2293,41 | 4,35 |
| sept | Port-Neuf | 8 | 0,01 | 103,86 | 60,60 | 1651,00 | 5,20 |
| sept | Port-Neuf | 9 | 0,02 | 147,34 | 60,37 | 2743,66 | 6,37 |
| sept | Port-Neuf | 10 | 0,02 | 159,39 | 56,86 | 2120,53 | 4,83 |
| sept | Minimes | 1 | 0,03 | 64,83 | 50,94 | NA | 3,11 |
| sept | Minimes | 2 | 0,03 | 92,98 | 51,66 | 1683,02 | 3,10 |
| sept | Minimes | 3 | 0,02 | 63,41 | 63,25 | 2158,37 | 3,36 |
| sept | Minimes | 4 | 0,03 | 118,77 | NA | 1972,01 | 3,26 |
| sept | Minimes | 5 | 0,03 | 106,67 | 48,33 | 1560,38 | 3,26 |
| sept | Minimes | 6 | 0,02 | 118,78 | 51,68 | 2219,62 | 2,92 |
| sept | Minimes | 7 | 0,01 | 99,61 | 40,94 | 1644,39 | 3,85 |
| sept | Minimes | 8 | 0,01 | 84,76 | 47,63 | 1767,04 | 3,21 |
| sept | Minimes | 9 | 0,02 | 68,75 | 53,15 | 1643,07 | 2,88 |
| sept | Minimes | 10 | 0,02 | 77,29 | 72,39 | 2236,24 | 3,50 |
| sept | Les Palles | 1 | 0,04 | 114,46 | 44,31 | 1842,44 | 4,24 |
| sept | Les Palles | 2 | 0,03 | 95,47 | 41,26 | 1259,58 | 3,94 |
| sept | Les Palles | 3 | 0,04 | 90,79 | 38,97 | NA | NA |
| sept | Les Palles | 4 | 0,02 | 88,18 | 39,89 | 1908,95 | NA |
| sept | Les Palles | 5 | 0,01 | 69,56 | 38,26 | 1278,28 | 4,59 |
| sept | Les Palles | 6 | 0,02 | 70,73 | 39,83 | 1426,58 | 4,64 |
| sept | Les Palles | 7 | 0,02 | 131,37 | 39,24 | 1358,33 | 2,73 |
| sept | Les Palles | 8 | 0,02 | 105,28 | 52,63 | 1918,12 | 3,31 |
| sept | Les Palles | 9 | 0,03 | 97,54 | 39,27 | 1775,46 | 3,05 |
| sept | Les Palles | 10 | 0,04 | 134,16 | 41,78 | 1895,29 | 3,38 |

**S2b**

| **date** | **sampling site** | **individual** | **As** | **Cd** | **Co** | **Cr** | **Cu** | **Fe** | **Mn** | **Ni** | **Pb** | **Se** | **V** | **Zn** | **Ag** |
| --- | --- | --- | --- | --- | --- | --- | --- | --- | --- | --- | --- | --- | --- | --- | --- |
| march | Loix | 1 | 14,91 | 16,25 | 0,62 | 1,05 | 22,77 | 476,73 | 19,22 | 2,11 | 9,43 | 7,69 | 2,39 | 60,05 | 5,74 |
| march | Loix | 2 | 15,04 | 21,52 | 0,56 | 0,74 | 14,19 | 346,83 | 13,65 | 1,61 | 2,83 | 8,19 | 1,71 | 65,80 | 3,94 |
| march | Loix | 3 | 16,37 | 19,76 | 0,72 | 1,83 | 34,87 | 863,11 | 26,14 | 2,81 | 8,00 | 12,85 | 3,65 | 75,52 | 9,94 |
| march | Loix | 4 | 18,32 | 40,21 | 0,74 | 1,18 | 60,47 | 418,76 | 14,75 | 9,42 | 6,78 | 11,42 | 2,95 | 77,68 | 4,58 |
| march | Loix | 5 | 19,79 | 20,32 | 0,67 | 0,64 | 21,45 | 378,28 | 22,15 | 1,15 | 2,36 | 10,17 | 1,43 | 71,53 | 7,12 |
| march | Loix | 6 | 17,94 | 35,13 | 0,83 | 1,41 | 55,52 | 406,64 | 16,00 | 2,66 | 4,80 | 10,87 | 3,76 | 55,97 | 5,04 |
| march | Loix | 7 | 19,70 | 25,49 | 0,80 | 1,12 | 36,45 | 310,75 | 19,21 | 1,88 | 3,10 | 9,47 | 2,17 | 61,93 | 13,81 |
| march | Loix | 8 | 18,36 | 24,09 | 0,69 | 1,18 | 36,66 | 414,26 | 16,60 | 2,77 | 4,89 | 10,95 | 2,91 | 67,54 | 8,08 |
| march | Loix | 9 | 17,28 | 29,26 | 0,75 | 1,30 | 31,83 | 442,16 | 15,58 | 2,24 | 5,02 | 11,27 | 3,26 | 68,24 | 12,74 |
| march | Loix | 10 | 17,22 | 18,16 | 0,49 | 1,00 | 16,17 | 376,29 | 14,28 | 1,26 | 4,09 | 9,86 | 1,99 | 72,02 | 5,67 |
| march | Port-Neuf | 1 | 15,25 | 61,59 | 0,73 | 1,59 | 54,60 | 361,29 | 17,93 | 2,46 | 5,75 | 12,03 | 1,59 | 203,77 | 7,37 |
| march | Port-Neuf | 2 | 15,53 | 50,80 | 1,07 | 2,23 | 54,30 | 620,50 | 19,88 | 1,86 | 4,79 | 11,79 | 1,43 | 136,51 | 10,12 |
| march | Port-Neuf | 3 | 17,24 | 37,80 | 0,79 | 1,69 | 62,46 | 635,86 | 19,72 | 1,73 | 5,41 | 11,43 | 1,75 | 92,68 | 10,46 |
| march | Port-Neuf | 4 | 15,51 | 44,26 | 0,80 | 1,85 | 64,58 | 663,08 | 23,86 | 1,74 | 5,26 | 11,28 | 1,66 | 90,19 | 12,49 |
| march | Port-Neuf | 5 | 16,87 | 57,62 | 1,04 | 2,08 | 83,76 | 638,43 | 21,38 | 2,97 | 7,94 | 12,42 | 3,22 | 106,17 | 6,85 |
| march | Port-Neuf | 6 | 14,41 | 31,53 | 1,09 | 2,91 | 55,05 | 1547,58 | 39,66 | 3,22 | 12,95 | 11,97 | 4,34 | 89,32 | 6,86 |
| march | Port-Neuf | 7 | 14,49 | 52,83 | 1,20 | 3,10 | 107,40 | 1311,85 | 44,21 | 4,93 | 13,51 | 13,81 | 3,97 | 190,62 | 7,40 |
| march | Port-Neuf | 8 | 14,58 | 28,87 | 0,84 | 2,35 | 63,23 | 814,67 | 26,94 | 2,36 | 9,26 | 11,77 | 2,86 | 89,58 | 9,81 |
| march | Port-Neuf | 9 | 12,95 | 30,05 | 0,96 | 5,41 | 36,59 | 1356,05 | 37,27 | 2,94 | 16,30 | 11,00 | 3,94 | 99,27 | 6,59 |
| march | Port-Neuf | 10 | 14,34 | 28,74 | 1,06 | 2,45 | 54,85 | 1131,08 | 30,95 | 3,41 | 9,28 | 14,16 | 3,71 | 100,01 | 10,67 |
| march | Minimes | 1 | 14,22 | 108,68 | 1,36 | 2,85 | 136,66 | 1032,75 | 48,70 | 2,97 | 13,76 | 11,45 | 4,13 | 106,64 | 8,49 |
| march | Minimes | 2 | 14,34 | 103,14 | 1,01 | 2,94 | 231,07 | 961,94 | 26,44 | 2,32 | 6,63 | 10,35 | 1,99 | 108,62 | 13,63 |
| march | Minimes | 3 | 15,09 | 83,23 | 1,01 | 2,03 | 145,03 | 360,09 | 13,20 | 2,85 | 5,67 | 12,63 | 1,73 | 93,17 | 7,27 |
| march | Minimes | 4 | 14,01 | 80,70 | 1,10 | 2,77 | 70,52 | 726,14 | 18,25 | 2,46 | 4,48 | 9,61 | 1,45 | 244,32 | 14,06 |
| march | Minimes | 5 | 11,63 | 75,04 | 0,79 | 1,96 | 145,27 | 422,07 | 11,08 | 1,57 | 4,89 | 9,93 | 0,84 | 89,36 | 9,47 |
| march | Minimes | 6 | 14,68 | 75,88 | 0,98 | 1,66 | 109,16 | 386,67 | 11,23 | 4,23 | 5,17 | 13,72 | 2,44 | 100,50 | 7,52 |
| march | Minimes | 7 | 17,02 | 77,14 | 0,84 | 1,94 | 339,70 | 317,31 | 11,68 | 3,12 | 6,17 | 13,69 | 1,20 | 98,02 | 9,52 |
| march | Minimes | 8 | 14,74 | 96,45 | 1,10 | 2,77 | 293,59 | 625,59 | 17,60 | 2,60 | 5,17 | 12,01 | 1,32 | 81,04 | 10,02 |
| march | Minimes | 9 | 12,85 | 37,47 | 0,56 | 1,43 | 34,23 | 363,20 | 10,93 | 1,06 | 4,77 | 12,80 | 0,57 | 101,47 | 7,53 |
| march | Minimes | 10 | 14,08 | 78,78 | 0,98 | 2,16 | 158,44 | 472,91 | 15,80 | 3,03 | 7,21 | 12,94 | 1,21 | 106,66 | 5,26 |
| march | Les Palles | 1 | 16,11 | 64,74 | 1,01 | 2,27 | 77,02 | 923,73 | 20,86 | 2,30 | 6,02 | 18,33 | 3,23 | 154,54 | 8,72 |
| march | Les Palles | 2 | 15,15 | 61,00 | 0,96 | 2,62 | 76,33 | 1186,76 | 29,64 | 3,97 | 9,94 | 15,09 | 4,38 | 126,83 | 5,32 |
| march | Les Palles | 3 | 16,22 | 47,53 | 0,81 | 2,11 | 40,39 | 1029,55 | 30,05 | 3,11 | 5,63 | 16,58 | 3,19 | 98,19 | 9,35 |
| march | Les Palles | 4 | 20,16 | 47,57 | 0,78 | 1,05 | 57,91 | 596,70 | 16,25 | 1,75 | 3,60 | 14,71 | 1,11 | 105,11 | 9,48 |
| march | Les Palles | 5 | 19,68 | 41,07 | 0,94 | 2,01 | 21,76 | 711,99 | 24,50 | 4,55 | 4,07 | 16,97 | 1,84 | 186,80 | 17,28 |
| march | Les Palles | 6 | 20,65 | 40,03 | 0,80 | 1,12 | 28,33 | 631,27 | 17,04 | 1,73 | 3,39 | 16,81 | 1,09 | 177,64 | 19,15 |
| march | Les Palles | 7 | 16,53 | 38,38 | 0,78 | 1,64 | 25,38 | 662,63 | 21,26 | 3,85 | 6,05 | 13,51 | 2,64 | 427,79 | 13,25 |
| march | Les Palles | 8 | 17,62 | 37,02 | 1,00 | 2,38 | 25,37 | 863,73 | 26,16 | 4,90 | 6,23 | 16,26 | 1,79 | 436,74 | 14,94 |
| march | Les Palles | 9 | 16,98 | 37,15 | 0,97 | 2,64 | 41,95 | 1189,27 | 32,22 | 2,61 | 5,68 | 16,77 | 2,12 | 102,04 | 12,75 |
| march | Les Palles | 10 | 20,84 | 36,96 | 0,95 | 3,20 | 28,47 | 1171,93 | 32,40 | 2,59 | 5,26 | 18,87 | 1,96 | 332,82 | 12,52 |
| sept | Loix | 1 | 10,18 | 11,56 | 0,56 | 0,36 | 16,39 | 681,72 | 15,24 | 0,99 | 0,96 | 6,35 | 2,85 | 55,46 | 3,91 |
| sept | Loix | 2 | 10,68 | 29,23 | 0,88 | 0,62 | 65,65 | 569,70 | 13,52 | 1,67 | 1,08 | 7,70 | 3,38 | 59,80 | 19,46 |
| sept | Loix | 3 | 8,82 | 13,63 | 0,50 | 0,47 | 18,30 | 597,24 | 12,95 | 0,90 | 0,92 | 4,58 | 2,15 | 47,38 | 4,35 |
| sept | Loix | 4 | 19,11 | 25,86 | 0,96 | 0,67 | 28,95 | 646,54 | 14,29 | 1,69 | 0,98 | 7,92 | 3,89 | 77,91 | 6,98 |
| sept | Loix | 5 | 12,34 | 29,52 | 0,79 | 0,65 | 31,02 | 469,05 | 8,23 | 1,18 | 1,00 | 5,69 | 2,79 | 75,70 | 9,00 |
| sept | Loix | 6 | 10,37 | 19,09 | 0,60 | 0,47 | 34,79 | 456,61 | 13,23 | 1,12 | 0,75 | 4,69 | 2,31 | 69,56 | 10,89 |
| sept | Loix | 7 | 9,08 | 12,11 | 0,49 | 0,26 | 14,93 | 586,64 | 10,24 | 1,10 | 1,07 | 4,72 | 1,99 | 43,29 | 6,28 |
| sept | Loix | 8 | 11,06 | 13,80 | 0,59 | 0,39 | 41,42 | 527,93 | 10,46 | 1,77 | 0,76 | 5,41 | 2,47 | 62,62 | 13,35 |
| sept | Loix | 9 | 18,46 | 37,67 | 1,01 | 0,84 | 34,30 | 494,31 | 11,62 | 2,01 | 0,96 | 9,63 | 4,42 | 66,79 | 13,13 |
| sept | Loix | 10 | 12,33 | 22,65 | 0,93 | 0,92 | 31,54 | 1093,71 | 17,23 | 1,77 | 1,44 | 5,75 | 4,44 | 73,89 | 7,48 |
| sept | Port-Neuf | 1 | 6,64 | 33,52 | 0,33 | 0,17 | 72,39 | 321,61 | 10,25 | 0,68 | 0,66 | 4,13 | 1,31 | 51,04 | 17,05 |
| sept | Port-Neuf | 2 | 9,46 | 43,31 | 0,62 | 0,80 | 90,67 | 650,53 | 20,88 | 1,57 | 1,25 | 4,54 | 2,90 | 91,24 | 18,00 |
| sept | Port-Neuf | 3 | 10,54 | 42,21 | 0,57 | 0,64 | 108,38 | 468,91 | 16,41 | 1,22 | 0,90 | 6,14 | 1,92 | 76,47 | 4,89 |
| sept | Port-Neuf | 4 | 8,21 | 40,96 | 0,51 | 0,39 | 72,23 | 388,24 | 10,73 | 0,73 | 0,75 | 5,10 | 1,43 | 63,50 | 29,13 |
| sept | Port-Neuf | 5 | 8,14 | 26,27 | 0,45 | 0,43 | 41,20 | 426,55 | 11,92 | 0,89 | 0,77 | 5,24 | 1,85 | 55,24 | 8,29 |
| sept | Port-Neuf | 6 | 11,12 | 31,83 | 0,61 | 0,38 | 45,87 | 435,45 | 12,02 | 1,19 | 0,92 | 5,96 | 2,42 | 68,28 | 10,79 |
| sept | Port-Neuf | 7 | 10,01 | 33,45 | 0,60 | 0,65 | 75,03 | 537,29 | 14,92 | 1,44 | 1,04 | 6,48 | 2,15 | 54,12 | 14,21 |
| sept | Port-Neuf | 8 | 7,84 | 16,09 | 0,44 | 0,63 | 31,26 | 738,52 | 23,64 | 0,98 | 1,09 | 5,40 | 1,97 | 48,25 | 9,58 |
| sept | Port-Neuf | 9 | 10,70 | 37,91 | 0,57 | 0,52 | 75,47 | 416,16 | 12,38 | 1,04 | 1,02 | 6,95 | 2,39 | 55,95 | 11,87 |
| sept | Port-Neuf | 10 | 8,00 | 15,31 | 0,35 | 0,40 | 52,97 | 417,14 | 16,14 | 0,87 | 0,84 | 5,56 | 1,36 | 47,25 | 10,98 |
| sept | Minimes | 1 | 10,94 | 35,96 | 0,57 | 0,68 | 131,30 | 589,33 | 22,20 | 0,87 | 1,15 | 6,53 | 2,04 | 84,03 | 12,17 |
| sept | Minimes | 2 | 9,77 | 36,22 | 0,52 | 0,56 | 116,85 | 386,23 | 13,47 | 1,06 | 0,71 | 5,80 | 1,91 | 60,52 | 13,53 |
| sept | Minimes | 3 | 6,49 | 20,27 | 0,42 | 0,80 | 47,22 | 763,01 | 21,72 | 0,81 | 0,97 | 3,57 | 2,01 | 40,62 | 6,16 |
| sept | Minimes | 4 | 9,08 | 41,13 | 0,72 | 1,27 | 88,74 | 1229,77 | 30,15 | 1,43 | 1,58 | 5,30 | 3,23 | 58,33 | 23,09 |
| sept | Minimes | 5 | 11,26 | 36,39 | 0,73 | 0,98 | 290,45 | 800,00 | 25,28 | 1,30 | 1,18 | 5,61 | 2,67 | 66,70 | 16,67 |
| sept | Minimes | 6 | 10,45 | 26,31 | 0,63 | 1,08 | 98,75 | 1275,38 | 35,93 | 1,33 | 1,57 | 5,07 | 3,36 | 62,33 | 18,82 |
| sept | Minimes | 7 | 9,22 | 15,96 | 0,54 | 0,90 | 53,42 | 853,53 | 26,63 | 1,26 | 1,10 | 5,46 | 2,50 | 60,33 | 14,12 |
| sept | Minimes | 8 | 8,81 | 31,51 | 0,62 | 1,31 | 113,40 | 1045,20 | 31,40 | 1,23 | 1,38 | 4,46 | 3,06 | 67,33 | 12,04 |
| sept | Minimes | 9 | 8,64 | 36,43 | 0,56 | 0,70 | 97,40 | 516,80 | 15,40 | 0,87 | 0,86 | 4,84 | 1,90 | 73,12 | 26,31 |
| sept | Minimes | 10 | 7,08 | 27,15 | 0,49 | 0,71 | 86,09 | 817,21 | 22,17 | 0,92 | 1,02 | 3,95 | 1,98 | 47,49 | 20,03 |
| sept | Les Palles | 1 | 8,24 | 20,52 | 0,36 | 0,59 | 23,29 | 846,92 | 20,55 | 0,58 | 1,25 | 4,81 | 2,84 | 48,57 | 9,07 |
| sept | Les Palles | 2 | 7,80 | 12,41 | 0,34 | 0,51 | 13,00 | 716,64 | 19,23 | 0,67 | 1,07 | 5,18 | 2,96 | 70,81 | 6,24 |
| sept | Les Palles | 3 | 8,28 | 25,29 | 0,38 | 0,58 | 19,67 | 717,03 | 15,85 | 0,64 | 1,34 | 5,35 | 2,74 | 55,06 | 8,15 |
| sept | Les Palles | 4 | 7,22 | 14,02 | 0,36 | 0,56 | 14,46 | 878,28 | 39,27 | 0,62 | 1,22 | 5,90 | 2,51 | 56,65 | 7,70 |
| sept | Les Palles | 5 | 11,46 | 27,97 | 0,45 | 0,43 | 23,52 | 555,76 | 16,92 | 0,66 | 1,12 | 6,25 | 3,24 | 73,24 | 12,91 |
| sept | Les Palles | 6 | 8,93 | 35,10 | 0,37 | 0,38 | 37,10 | 501,34 | 13,13 | 0,69 | 0,96 | 6,14 | 2,84 | 57,30 | 31,73 |
| sept | Les Palles | 7 | 7,96 | 20,54 | 0,36 | 0,71 | 16,97 | 890,18 | 20,13 | 0,80 | 1,27 | 5,24 | 2,58 | 44,12 | 7,30 |
| sept | Les Palles | 8 | 7,94 | 20,58 | 0,34 | 0,27 | 17,43 | 530,41 | 19,49 | 0,49 | 1,02 | 5,32 | 2,54 | 123,25 | 8,69 |
| sept | Les Palles | 9 | 9,02 | 16,52 | 0,42 | 0,77 | 17,35 | 986,01 | 24,77 | 0,79 | 1,40 | 6,05 | 2,78 | 52,62 | 12,94 |
| sept | Les Palles | 10 | 7,62 | 15,18 | 0,31 | 0,56 | 13,24 | 668,02 | 18,47 | 0,50 | 1,00 | 4,91 | 2,54 | 69,71 | 7,12 |
