# Supplementary material for: Short-Term and Long-Term Biological Effects of Chronic Chemical Contamination on Natural Populations of a Marine Bivalve
Source: PLoS One. 2016 Mar 3;11(3):e0150184. doi: 10.1371/journal.pone.0150184 (PMC4777565; doi:10.1371/journal.pone.0150184)
Supplement: S3 Table — (DOCX) [file pone.0150184.s004.docx]

**Breitwieser, Viricel *et al.* - Supporting Information**

**S3 Table. Individual phosphatase activity data.**

| **date** | **sampling site** | **replicate number** | **phosphatase** |
| --- | --- | --- | --- |
| march | Loix | 1 | 0,036 |
| march | Loix | 2 | 0,076 |
| march | Loix | 3 | 0,077 |
| march | Port-Neuf | 1 | 0,041 |
| march | Port-Neuf | 2 | 0,058 |
| march | Port-Neuf | 3 | 0,060 |
| march | Minimes | 1 | 0,217 |
| march | Minimes | 2 | 0,362 |
| march | Minimes | 3 | 0,393 |
| march | Les Palles | 1 | 0,029 |
| march | Les Palles | 2 | 0,021 |
| march | Les Palles | 3 | 0,023 |
| sept | Loix | 1 | 0,255 |
| sept | Loix | 2 | 0,231 |
| sept | Loix | 3 | 0,230 |
| sept | Port-Neuf | 1 | 0,277 |
| sept | Port-Neuf | 2 | 0,318 |
| sept | Port-Neuf | 3 | 0,261 |
| sept | Minimes | 1 | 0,291 |
| sept | Minimes | 2 | 0,211 |
| sept | Minimes | 3 | 0,221 |
| sept | Les Palles | 1 | 0,302 |
| sept | Les Palles | 2 | 0,281 |
| sept | Les Palles | 3 | 0,283 |
